# Loss of *Slc12a2* specifically in pancreatic β-cells drives metabolic syndrome in mice

**DOI:** 10.1371/journal.pone.0279560

**Published:** 2022-12-29

**Authors:** Rana Abdelgawad, Yakshkumar Dilipbhai Rathod, Modhi Alshammari, Lisa Kelly, Christian A. Hübner, Lydia Aguilar-Bryan, Mauricio Di Fulvio

**Affiliations:** 1 Department of Pharmacology and Toxicology, Wright State University, School of Medicine Dayton, Fairborn, Ohio, United States of America; 2 Institut für Humangenetik, Universitätsklinikum Jena, Jena, Germany; 3 Pacific Northwest Diabetes Research Institute, Seattle, Washington, United States of America; Tohoku University, JAPAN

## Abstract

The risk of type-2 diabetes and cardiovascular disease is higher in subjects with metabolic syndrome, a cluster of clinical conditions characterized by obesity, impaired glucose metabolism, hyperinsulinemia, hyperlipidemia and hypertension. Diuretics are frequently used to treat hypertension in these patients, however, their use has long been associated with poor metabolic outcomes which cannot be fully explained by their diuretic effects. Here, we show that mice lacking the diuretic-sensitive Na^+^K^+^2Cl^−^cotransporter-1 *Nkcc1* (*Slc12a2*) in insulin-secreting β-cells of the pancreatic islet (*Nkcc1*^*βKO*^) have reduced *in vitro* insulin responses to glucose. This is associated with islet hypoplasia at the expense of fewer and smaller β-cells. Remarkably, *Nkcc1*^*βKO*^ mice excessively gain weight and progressive metabolic syndrome when fed a standard chow diet *ad libitum*. This is characterized by impaired hepatic insulin receptor activation and altered lipid metabolism. Indeed, overweight *Nkcc1*^*βKO*^ but not lean mice had fasting and fed hyperglycemia, hypertriglyceridemia and non-alcoholic steatohepatitis. Notably, fasting hyperinsulinemia was detected earlier than hyperglycemia, insulin resistance, glucose intolerance and increased hepatic *de novo* gluconeogenesis. Therefore, our data provide evidence supporting the novel hypothesis that primary β-cell defects related to *Nkcc1*-regulated intracellular Cl^−^homeostasis and β-cell growth can result in the development of metabolic syndrome shedding light into additional potential mechanisms whereby chronic diuretic use may have adverse effects on metabolic homeostasis in susceptible individuals.

## Introduction

Metabolic syndrome (MetS) is a common cluster of metabolic conditions reaching epidemic proportions. The main features include overweight/obesity, impaired glucose metabolism, hyperinsulinemia, hyperlipidemia and hypertension, which together strongly increase the risk for cardiovascular disease (CVD) and type-2 diabetes (T2D) [1–3]. In fact, the MetS is more frequent than T2D and its prevalence increases with age and overweight [4]. In addition, MetS together with obesity is considered the primary cause of non-alcoholic fatty liver disease (NAFLD) and its complications [5]. Therefore, preventing MetS constitutes a fundamental strategy to reduce CVD and T2D prevalence [1].

The etiology of the metabolic syndrome is complex. However, it is generally accepted that overeating calorie-dense diets rich in fats [6] and/or a sedentary life-style [7], is what eventually leads to increased adiposity, ectopic fat deposits, low-grade tissue inflammation, overweight/obesity and insulin resistance, the main drivers of the syndrome [8]. Current evidence suggests that insulin responses to feeding also play a role in the acute control of food intake [9] and that chronic hyperinsulinemia secondary to abnormal secretion/clearance is associated with a rise in fat mass accumulation [10]. It has been shown that lean individuals at risk of developing obesity have characteristically high and/or dynamically different insulin responses to nutrients, which persist or worsen during obesity [11, 12]. Moreover, obese individuals also show abnormal pulsatile insulin secretion [13, 14], all consistent with the notion that primary functional deficiencies in the islet secretory response to nutrients can contribute to the development of overweight and its complications including the MetS and T2D [15, 16].

It is well recognized that pancreatic β-cells release insulin in a pulsatile manner [17] and in synchrony with intracellular Ca^2+^ and/or metabolic oscillations [18, 19]. Particularly, some of the mechanisms proposed to underlie β-cell Ca^2+^/metabolic oscillations and electrical bursting [20] appear unrelated to the canonical K_ATP_ channel [21–25]. Indeed, a wide range of insulinotropic glucose concentrations promotes electrogenic Cl^−^fluxes while K_ATP_ channel activity remains inhibited [26, 27] whereas blocking these Cl^−^currents abolished membrane potential and Ca^2+^ oscillations [28–31]. Chloride fluxes do require Cl^−^channels and some of them were independently implicated in β-cell function. For instance, volume-regulated anion channels (VRAC) [32–34], anoctamine-1 (*Ano1*) [30, 31], the cystic fibrosis transmembrane conductance regulator (*Cftr*) [30, 35] or the ionotropic receptors for γ-aminobutyric acid (GABA) [36, 37] and glycine [38] all participate, to different extents, in β-cell excitability and insulin secretion. Independent of which Cl^−^channels are involved, secondary active Cl^−^loaders and extruders determine the non-equilibrium distribution of the anion and set the driving force for Cl^−^to flux through channels [39]. Inhibition of Cl^−^loaders such as *Nkcc1* (*Slc12a2*) and others (*Nkcc2*, *Slc12a1*) with loop-diuretics bumetanide or furosemide impaired islet insulin secretion *in vitro* and resulted in glucose intolerance in different mouse models [40–44]. In addition, we have recently demonstrated that mice lacking a variant of the bumetanide-sensitive *Nkcc2* (*Nkcc2a*, *Slc12a1v1*) exhibit abnormal insulin responses to glucose and develop hyperglycemia, glucose intolerance and insulin resistance [45]. In humans, diuretic treatment has been long associated with altered glucose homeostasis, insulin resistance [46–52] and worsening of the MetS [53]. Further, patients with functional deficiency of the thiazide-sensitive Cl^−^loader *SLC12A3* are prone to overweight/obesity and the MetS [54–58]. At this point, it is important to keep in mind that the targets of diuretics, including *Nkcc1*, *Nkcc2a* and *Slc12a3* are highly expressed in the kidney when compared to pancreatic β-cells [59, 60] and that diuretics can inhibit islet insulin secretion directly [61–63]. Therefore, the diuretic-dependent worsening of metabolic homeostasis may, at least in part be mediated by extra-renal effects of these drugs.

Here, we generated a new mouse model constitutively lacking the *Nkcc1* in β-cells (*Nkcc1*^*βKO*^) and present experimental evidence indicating spontaneous development of typical features of the metabolic syndrome in these mice. Indeed, by 30 weeks of age *Nkcc1*^*βKO*^ mice fed *ad libitum* a standard diet, are overweight, glucose intolerant, insulin resistant and develop non-alcoholic steatohepatitis (NASH). Therefore, our results provide a potential mechanistic explanation for the metabolic disturbances provoked by the chronic use of diuretics and a new pre-clinical mouse model to study the spontaneous development and progression of a syndrome considered a major risk factor of CVD and T2D.

## Results

### Effective and specific elimination of *Nkcc1* in pancreatic β-cells

**Fig 1:**
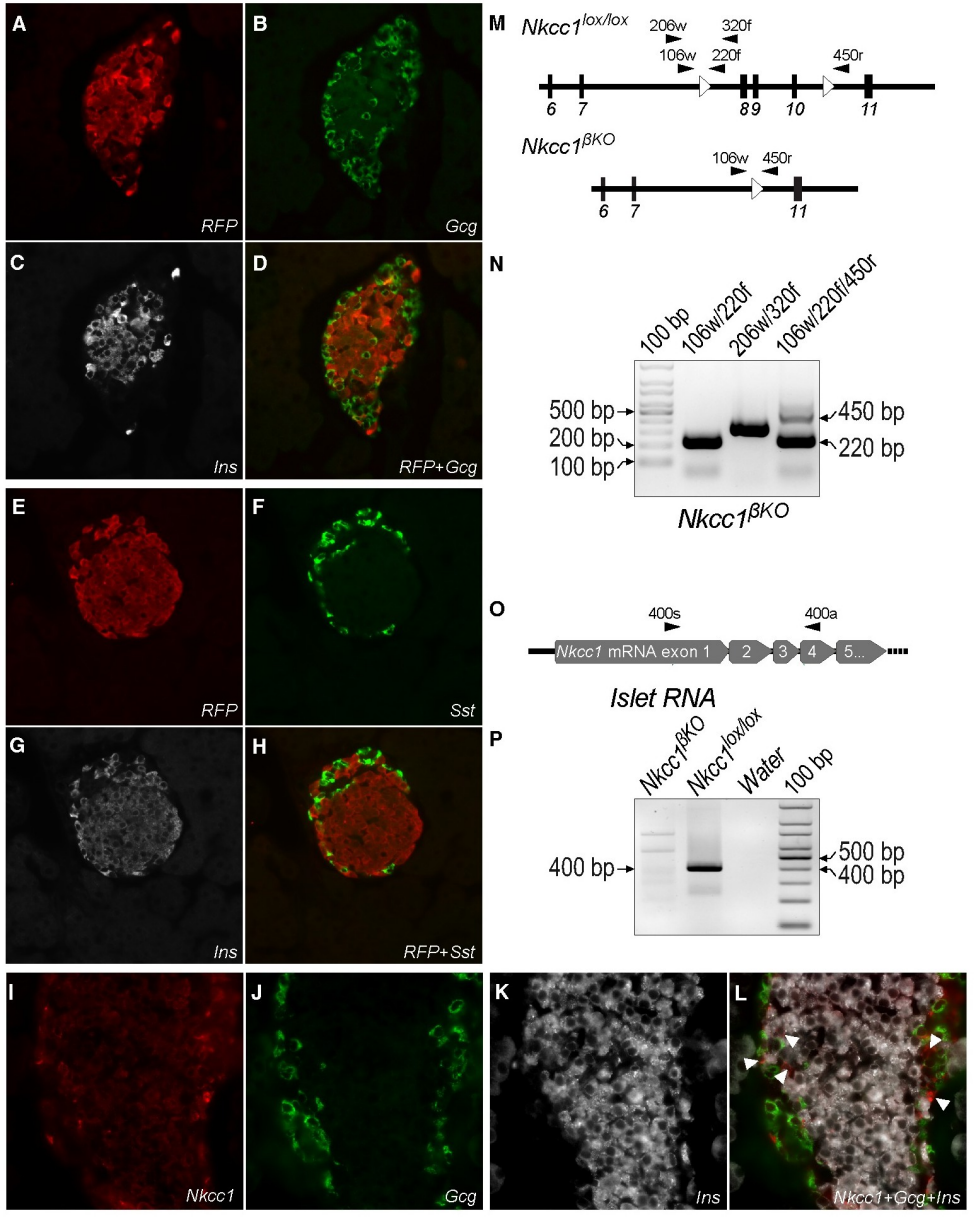
The *Ins1*^*Cre*^ line deletes target alleles exclusively in β-cells of the pancreatic islet. **A-H**. Representative pancreas sections of *Ins1*^*Cre*^:*Nkcc1*^*lox/lox*^:*Tomato* mice. Islets were coimmunolabeled against *RFP* (A and E), insulin (*Ins*, C and G), glucagon (*Gcg*, B) and somatostatin (*Sst*, F) to identify β-, α- or δ-cells, respectively. Overlay images of A-C and E-G are shown in D and H, respectively. **I-L**. Representative pancreas sections of *Nkcc1*^*βKO*^ mice coimmunolabeled against *Nkcc1* (I), *Gcg* (J) and *Ins* (K) showing β-cell-specific *Nkcc1* deletion in the overlay image (L). Arrowheads in L indicate *Nkcc1* immunoreactivity in *Ins*- or *Gcg*-negative cells. **M**. Shown are exons 6–11 (filled boxes) of the mouse *Slc12a2* gene and *Lox* sites (empty arrowheads). Filled arrowheads indicate PCR primers 106w/220f and 206w/320f designed to amplify 5’ *Lox* sites as 220bp and 320bp bands, respectively. The primer triplet 106w/220f/450r co-amplifies 220bp and 450bp fragments corresponding to the *Nkcc1*^*lox/lox*^ genotype of non-β cells and *Cre*/*Lox*-recombined alleles of β-cells, respectively. **N**. PCR of islet genomic DNA from *Nkcc1*^*βKO*^ mice showing amplicons of expected sizes by using the primers indicated in M. **O**. Represented are exons 1–5 (filled arrows) of *Nkcc1* mRNAs and the RT-PCR primer pair 400s/400a used to produce *Nkcc1* amplicons of 400bp. **P**. Representative RT-PCR experiments using total islet RNA from *Nkcc1*^*lox/lox*^ and *Nkcc1*^*βKO*^ mice. *Nkcc1* mRNA expression detected as amplicons of expected sizes mainly in *Nkcc1*^*lox/lox*^ samples.

To ascertain β-cell specific *Cre*-mediated recombination, we determined immunoreactive red-fluorescent protein (*RFP*) and *Nkcc1* expression in pancreatic islets and brain tissue dissected from *Ins1*^*Cre*^:*Nkcc1*^*lox/lox*^:*Tomato* reporter mice, using immunofluorescence microscopy. For these experiments, we used *Nkcc1* antibodies validated against *Nkcc1*^*KO*^ tissues (S1A–S1H Fig). As expected, 15w old *Ins1*^*Cre*^:*Nkcc1*^*lox/lox*^:*Tomato* mice expressed RFP in insulin-positive cells (Fig 1A and 1E) but not in glucagon (Fig 1B and 1D) or somatostatin cells (Fig 1F and 1H) demonstrating β-cell-specific *Cre*-mediated recombination. In addition, 25w old *Nkcc1*^*βKO*^ mice did not show immunoreactivity for *Nkcc1* in islet β-cells (Fig 1I–1L) but was present in glucagon-negative cells (arrowheads in Fig 1L). Consistent with previous data [64], *Nkcc1* was barely detected in *Nkcc1*^*βKO*^ α-cells (Fig 1L) or control *Ins1*^*Cre*^ or *Nkcc1*^*lox/lox*^ (S1E–S1H and S2A–S2H Figs). Consistently, PCR and RT-PCR experiments demonstrate the expected genomic recombination event (Fig 1M and 1N) and undetectable *Nkcc1* transcripts (Fig 1O and 1P) in islets from 25w old *Nkcc1*^*βKO*^ mice. Moreover, *Nkcc1* protein expression was intact in the choroid plexus and in the brain of *Ins1*^*Cre*^:*Nkcc1*^*lox/lox*^:*Tomato* (S1I Fig) whereas *Cre* expression or *"floxed" Slc12a2* alleles *per se* did not alter *Nkcc1* expression patterns in the islet or tissues of 15w old *Ins1*^*Cre*^ and *Nkcc1*^*lox/lox*^ mice (S2 Fig). Therefore, these results suggest that *Nkcc1*^*βKO*^ mice lack *Nkcc1* expression only in insulin-secreting β-cells.

### Loss of *Nkcc1* in β-cells reduces β-cell mass, insulin secretion and action

**Fig 2:**
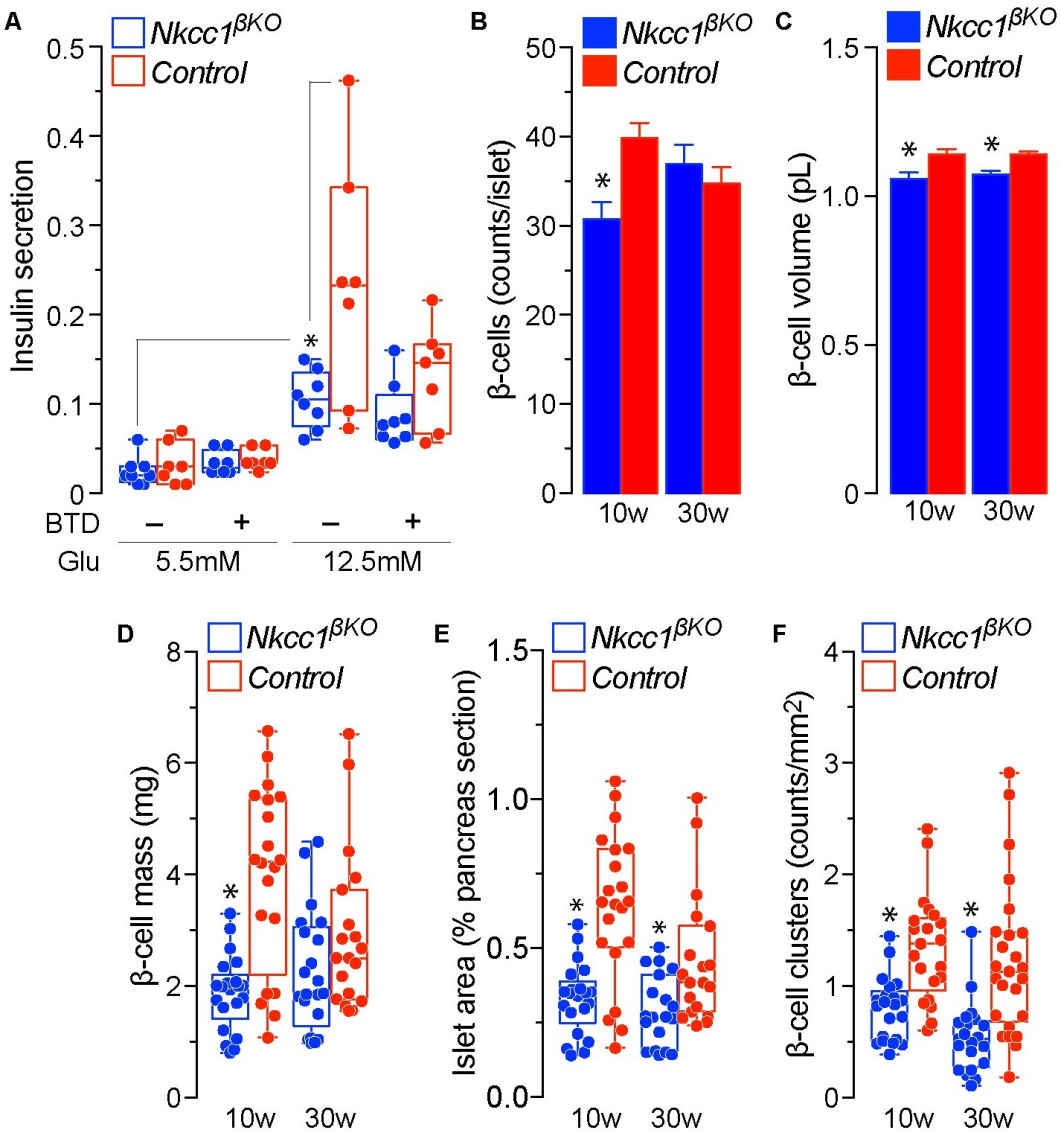
Loss of *Nkcc1* in β-cells reduces islet insulin secretion and β-cell mass. **A**. Insulin secretory responses to low (5.5mM) and high (12.5mM) glucose of islets from 22w old *Nkcc1*^*βKO*^ and control mice (*Nkcc1*^*lox/lox*^) in the presence of vehicle (DMSO) or 10μM bumetanide (BTD), as indicated. Results are expressed as the mean ± SEM of insulin secreted relative to total islet insulin content (*n* = 7–8, *p<0.05). **B-F**. Morphometry analysis performed on pancreas sections from *Nkcc1*^*βKO*^ and control mice (*Nkcc1*^*lox/lox*^) at the indicated ages and immunolabeled against insulin. Shown are the number of β-cells per islet (B), mean β-cell volume (C, pL), β-cell mass (D, mg), islet area (E, % pancreas section) and the number of β-cell clusters (representing ≤5 β-cells/cluster) per mm^2^ of pancreas tissue section (F). The data in B-C represents the mean ± SEM corresponding to >700 individual islets identified in 19–21 tissue sections obtained from male mice (*n* = 3) of the indicated genotypes and ages. Each point in D-F represents the mean values per single tissue section (*p<0.05).

The results shown in Fig 2A demonstrate that islets from ~22w old *Nkcc1*^*βKO*^ mice are less responsive to glucose than control islets (*Ins1*^*Cre*^). Importantly, bumetanide reduced the secretory response to glucose in control but not in *Nkcc1*^*βKO*^ islets, as expected for a highly specific inhibitor of *Nkcc1* and *Nkcc2*. These data thus confirm functional elimination of the transporter in β-cells of *Nkcc1*^*βKO*^ islets. Note that the secretory response of islets from 8-10w old *Nkcc1*^*βKO*^ mice was reduced, albeit not significantly (S3A Fig). To determine if these findings relate to changes in islet β-cell number/size, a morphometric analysis was performed. The data demonstrates significantly reduced β-cell numbers (Fig 2B), volume (Fig 2C) and mass (Fig 2D) in 10w old *Nkcc1*^*βKO*^ relative to age-matched control mice (*Nkcc1*^*lox/lox*^). Accordingly, the islet-to-pancreas area ratio was significantly reduced in 10w old *Nkcc1*^*βKO*^ (Fig 2E) as well as the total number of β-cell clusters throughout the *Nkcc1*^*βKO*^ pancreas (Fig 2F). As expected for normal mice, the β-cell morphometry parameters obtained in 10w old mice remained relatively unchanged in older mice. Since there were no significant differences found in α-cell count, volume, mass or area between mice of both genotypes (S3 Fig), together these data suggest that a combination of reduced β-cell volume and number contribute to the reduced *Nkcc1*^*βKO*^ islet secretory responses *in vitro* and overall reduction in pancreatic β-cell mass in *Nkcc1*^*βKO*^ mice.

**Fig 3:**
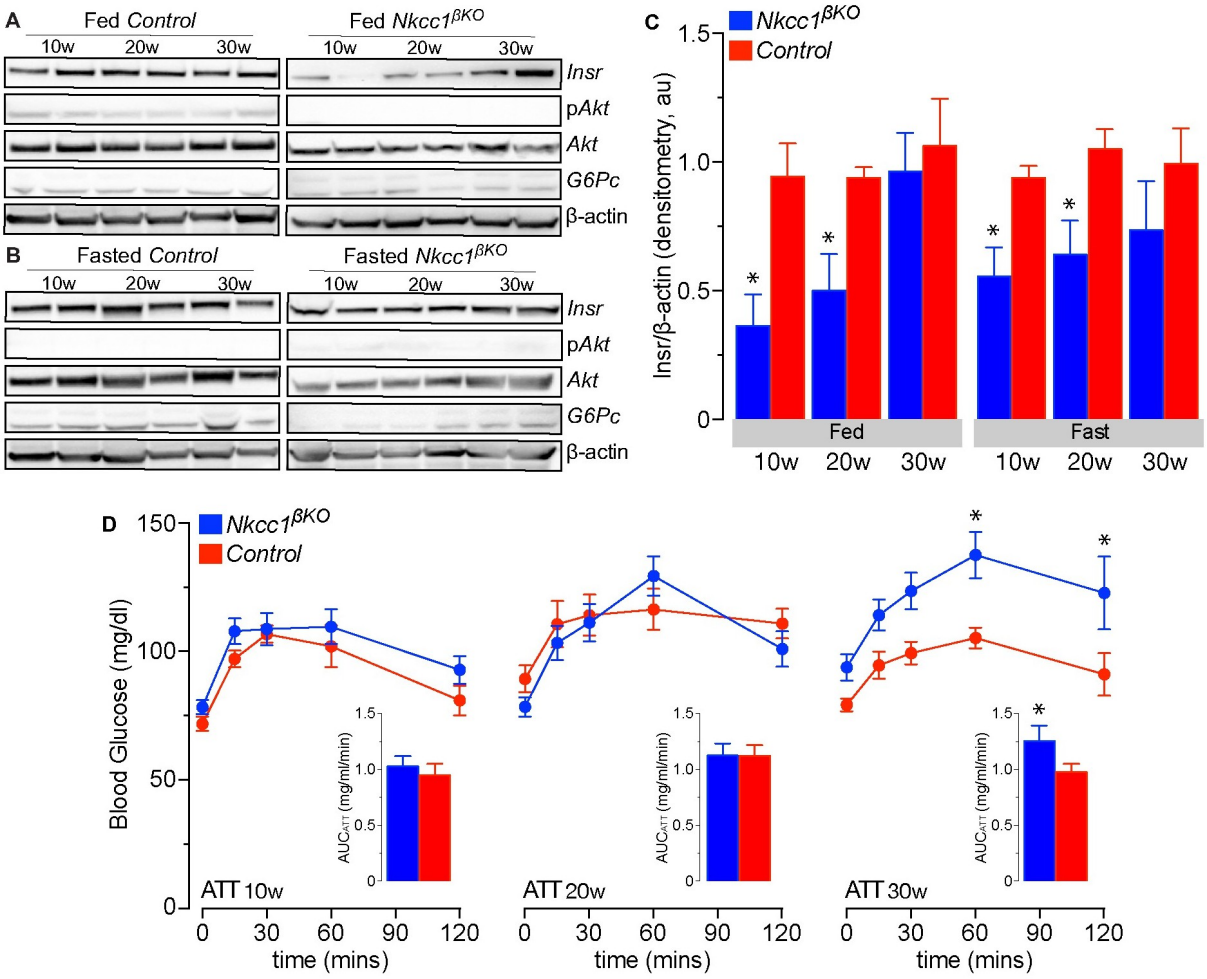
Hepatic insulin receptor expression, signaling and de novo gluconeogenesis in *Nkcc1*^*β*^KO mice. **A, B**. Expression pattern of insulin receptors (*Insr*, 95kDa), *Akt* (60kDa) and *G6Pc* (40kDa) and phospho-activation of *Akt* (p*Akt*) in liver extracts of 10w, 20w and 30w *Nkcc1*^*βKO*^ and control mice (*Ins1*^*Cre*^) fed (A) or fasted 16h (B). Shown are representative immunoblots loaded to represent 2 mice (*n* = 3–4 per genotype, age and condition). As loading control, we used β-actin (45kDa). **C**. Semi-quantitative densitometry analysis of hepatic *Insr* expression levels relative to β-actin expressed in arbitrary units (*au*). Shown are the mean ± SEM of 3 independent blots corresponding to 3 male mice of the indicated genotypes, ages and condition (*p<0.05). **D**. Blood glucose excursions (mg/dl) during alanine tolerance tests (ATT) performed in 16h fasted *Nkcc1*^*βKO*^ and control mice (*Ins1*^*Cre*^) at the indicated ages (mean ± SEM, *n* = 9–10, *p<0.05). The areas under each curve (mg/ml/min) are indicated as insets in D.

Since normal insulin secretion activates hepatic insulin signaling to reduce *de novo* gluconeogenesis [65], we evaluated age-dependent hepatic insulin receptor (*Insr*)-mediated *Akt* phosphorylation and *G6Pc* expression in fed and 16h fasted 10-30w old *Nkcc1*^*βKO*^ mice. Fed control mice (*Ins1*^*Cre*^) showed the expected *Insr*-mediated increase in *Akt* phosphorylation (Fig 3A, *left panel*), which was barely detected in *Nkcc1*^*βKO*^ mice at all ages tested (Fig 3A, *right panel*). Thus, in *Nkcc1*^*βKO*^ mice the response of the liver to food intake appears blunted. When mice were fasted, *Akt* phosphorylation was neither detected in control mice, as expected, nor in *Nkcc1*^*βKO*^ (Fig 3B). These data indicate reduced post-prandial hepatic *Insr* signaling in *Nkcc1*^*βKO*^ mice. However, expression levels of *Insr* were found reduced only in younger (10-20w) *Nkcc1*^*βKO*^ relative to controls and did not differ at 30 weeks of age in *Nkcc1*^*βKO*^ mice (Fig 3C). Interestingly, *G6Pc* protein expression relative to β-actin remained unchanged in *Nkcc1*^*βKO*^ mice suggesting intact endogenous glucose production. However, as shown in Fig 3D, glucose responses to exogenous alanine increased in 30w old *Nkcc1*^*βKO*^ mice, thus suggesting age-dependent deterioration in the control of hepatic *de novo* gluconeogenesis.

### Excess weight, increased fat mass and adipocyte hypertrophy in *Nkcc1*^*βKO*^ mice

**Fig 4:**
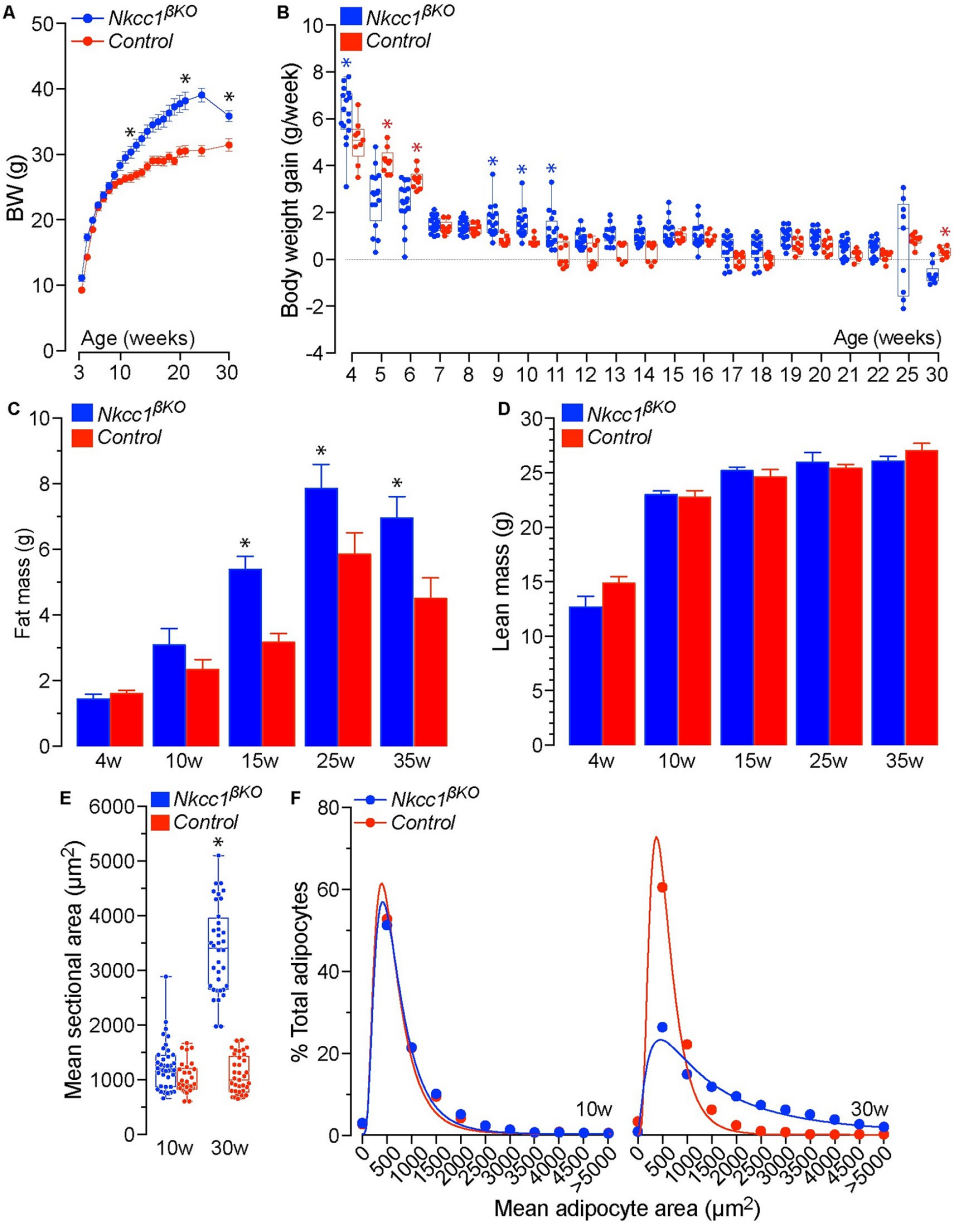
Absolute BW, gain, composition and adipose tissue morphometry of *Nkcc1*^*β*^KO mice. **A**. Growth of *Nkcc1*^*βKO*^ and control (*Nkcc1*^*lox/lox*^) mice fed *ad libitum* a chow diet. Data recorded as net weekly BW mass (g) starting at weaning until mice reached 30w of age. Plotted are the mean ± SEM (*n* = 9–16, *p<0.01). **B**. Weekly BW gain (g/week) of *Nkcc1*^*βKO*^ and control (*Nkcc1*^*lox/lox*^) mice computed by subtracting BW at a given week age to that of the previous week. Each point represents data from a single mouse (*n* = 9–16, *p<0.01). **C, D**. Indicated are the mean ± SEM values corresponding to net fat mass (C, g) and lean mass (D, g) of *Nkcc1*^*βKO*^ and control (*Nkcc1*^*lox/lox*^) mice at the indicated ages (*n* = 9–16, *p<0.01). **E**. Mean cross sectional area (μm^2^) of adipocytes morphometrically determined by analyzing retroperitoneal white fat tissue sections from 10w and 30w old *Nkcc1*^*βKO*^ and control (*Nkcc1*^*lox/lox*^) mice (*n* = 3). Each point represents the mean adipocyte area found in a single non-overlapping digital image randomly taken from tissue sections (*n* = 6–9) of the indicated genotypes and ages (*p<0.001). **F**. Relative mean adipocyte size distribution computed from the data in E.

*Ad libitum* chow-fed *Nkcc1*^*βKO*^ male mice significantly increased their body weight (BW) as they became older (Fig 4A), and this was not attributed to increased daily food intake (S4A Fig). Notably, BW mass of *Nkcc1*^*βKO*^ did not significantly differ from that of control mice (*Nkcc1*^*loxflox*^ or *Ins1*^*Cre*^) from weaning (p19-21) up to ~15w of age. Subsequently, *Nkcc1*^*βKO*^ were significantly heavier than control mice, without becoming overtly obese. As expected, weekly BW gain after weaning gradually declined with age in mice of both genotypes (Fig 4B). However, the initial reduction in post-weaning BW gain of *Nkcc1*^*βKO*^ was followed by an episodic burst of accelerated BW gain, which preceded the onset of BW mass increase. Indeed, BW decline was significantly faster in *Nkcc1*^*βKO*^ mice during the first 6w of age. After that, BW gain increased significantly during the 9^th^-11^th^w of age and remained hastened thereafter, but this significant difference disappeared over time relative to control mice. Notably, an increasing proportion of *Nkcc1*^*βKO*^ mice began to lose weight between 25w and 30w of age (Fig 4B) while their food intake also declined (S4A Fig). The results shown in Fig 4C and 4D confirm that *Nkcc1*^*βKO*^ BW accrual is due to a significant age-dependent increase in fat mass accumulation (Fig 4C) rather than lean mass (Fig 4D) or free/total body water content (S4B and S4C Fig). In a very consistent way, cross-sectional adipocyte areas and fat cell-size distribution in in white retroperitoneal fat tissue of 10w old *Nkcc1*^*βKO*^ mice were normal (Fig 4E and 4F, *left panel*). However, the mean adipocyte area and cell-size distribution were significantly expanded (Fig 4E), or shifted toward larger adipocytes, respectively, in 30w old *Nkcc1*^*βKO*^ mice (Fig 4F, right panel). In fact, 90–95% of the adipocytes were smaller than ~2000μm^2^ in 10w and 30w old normal mice whereas ~50% of all adipocytes in 30w old *Nkcc1*^*βKO*^ mice were larger than 2000μm^2^ (Fig 4F, *left panel*). Further, histological analysis of retroperitoneal white adipose tissue and pancreas of *Nkcc1*^*βKO*^ mice demonstrate infiltration of inflammatory cells (S4D and S4E Fig) and fat cell deposits (S4F and S4G Fig). Evidently, this increased local and ectopic fat mass accumulation and adipocyte hypertrophy account for the age-dependent increase in BW mass in *Nkcc1*^*βKO*^ mice.

### Dyslipidemia and non-alcoholic fatty liver disease in *Nkcc1*^*βKO*^ mice

**Fig 5:**
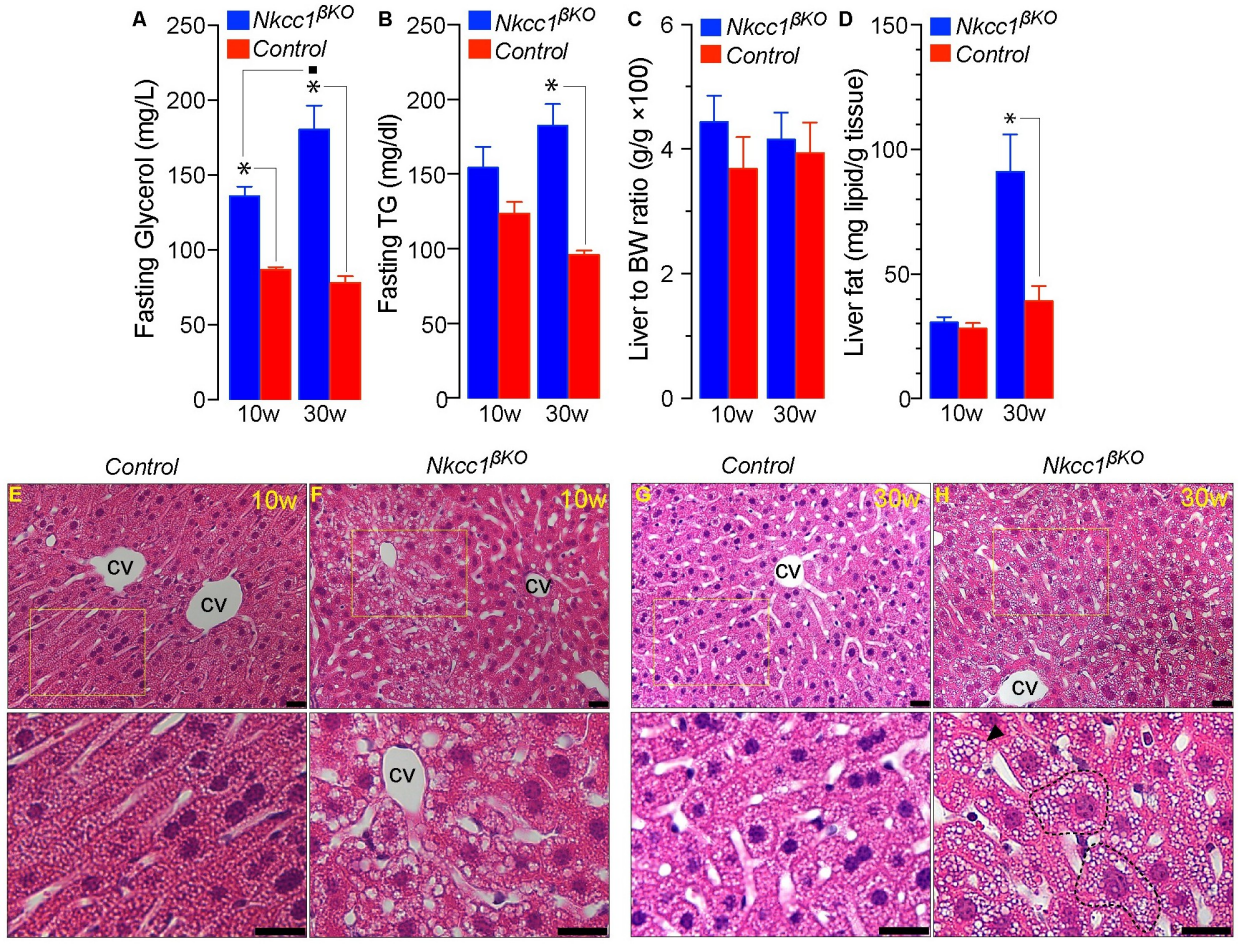
Plasma lipids, hepatic index, liver fat content and liver histopathology of *Nkcc1*^*β*^KO mice. **A, B**. Plasma glycerol (A, mg/L) and triglycerides (B, TG mg/dl) of 10w and 30w old *Nkcc1*^*βKO*^ and control (*Ins1*^*Cre*^) mice fasted 16h. Results represent the mean ± SEM (*n* = 4–5, *p<0.01). **C, D**. Plotted are the hepatic index (C) calculated as wet liver mass (g) relative to total BW (g), and the net fat content (mg) per gram of liver tissue (D) of *Nkcc1*^*βKO*^ and control (*Nkcc1*^*lox/lox*^) mice at the indicated ages. Results are expressed as the mean ± SEM (*n* = 5–6, *p<0.001). **E-H**. Shown are representative H&E-stained liver sections of 10w (E-F) or 30w old (G-H) control (*Nkcc1*^*lox/lox*^, E and G) and *Nkcc1*^*βKO*^ (F and H) mice. The squares in E-H are shown magnified in the images below each one of them to depict histopathology changes including mild steatosis around a central vein (cv) in 10w old *Nkcc1*^*βKO*^ mice and hypertrophic hepatocytes (dashed-lined cells), micro- and macro-vesicular fat deposits in 30w *Nkcc1*^*βKO*^ mice, consistent with a more severe steatosis phenotype (see S4 Fig). Bars indicate 20μm.

The results shown in Fig 5A demonstrate that plasma glycerol levels were significantly increased in 10w and 30w old *Nkcc1*^*βKO*^, but hypertriglyceridemia only manifested later in 30w old *Nkcc1*^*βKO*^ mice (Fig 5B) suggesting age-related deterioration of lipid metabolism. Importantly, *Nkcc1*^*βKO*^ did not develop larger livers than control mice discarding hepatomegaly (Fig 5C). Within this context, total fat content was significantly elevated in the liver of 30w old *Nkcc1*^*βKO*^ relative to control (Fig 5D, ~9% and ~4% w/w, respectively, *p<0.001) but not in 10w old *Nkcc1*^*βKO*^ mice (~3% w/w). Histological analysis revealed minimal and isolated micro vesicular steatosis in 10w old *Nkcc1*^*βKO*^ mice (Fig 5E and 5F) consistent with a normal score of 1 in the Kleiner’s scale of NAFLD [66]. However, 30w old *Nkcc1*^*βKO*^ showed hepatocyte hypertrophy, micro/macro vesicular steatosis (Fig 5G and 5H) and clusters of inflammatory cells, hepatocyte fat degeneration, rare cell ballooning (S5A–S5F Fig) and variable loss of hepatocyte glycogen content (S5G and S5H Fig). Therefore, 30w old *Nkcc1*^*βKO*^ mice developed non-alcoholic steatohepatitis (NASH).

### Age-dependent worsening of glycemic control in *Nkcc1*^*βKO*^ mice

**Fig 6:**
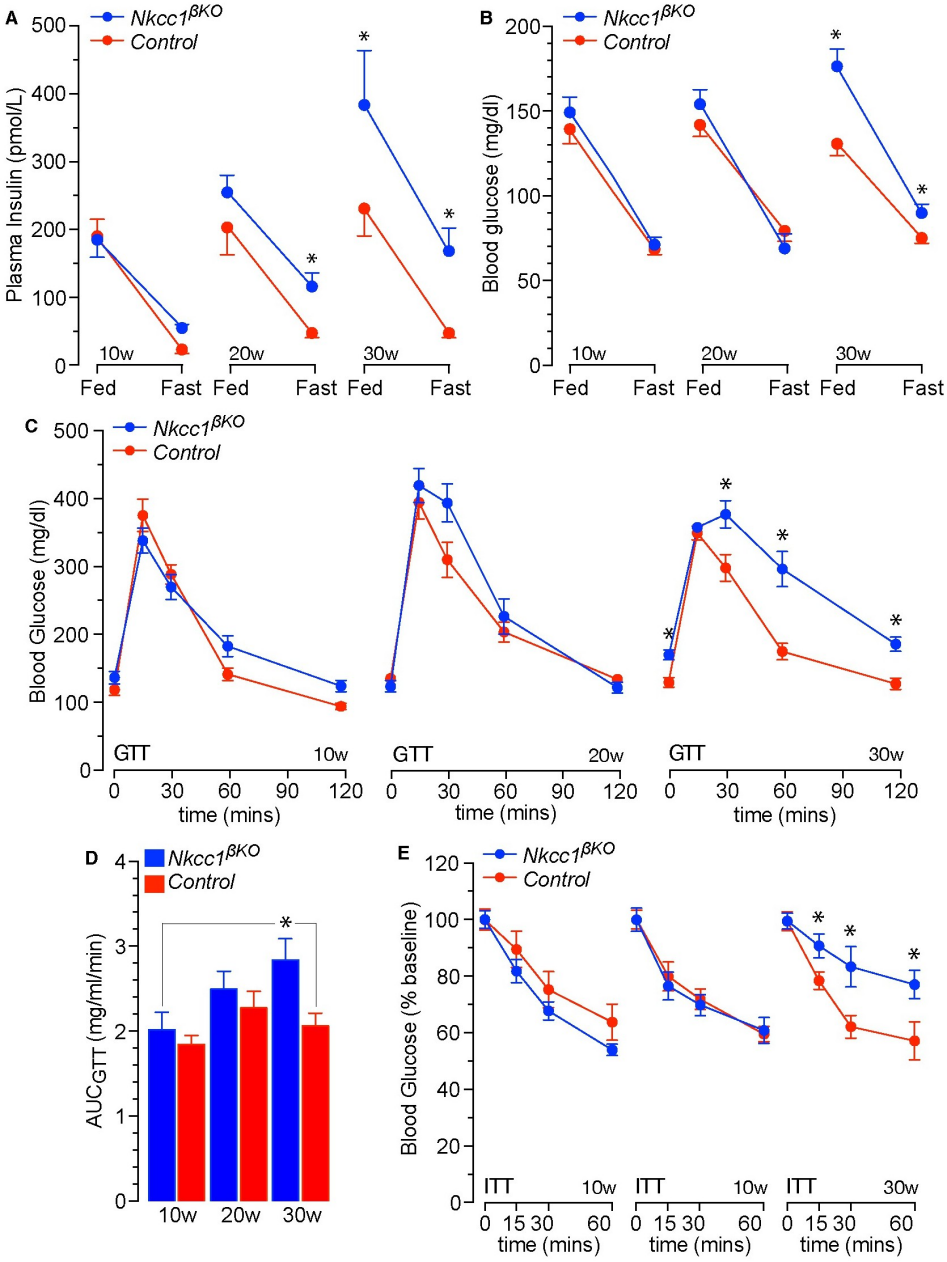
Plasma insulin, blood glucose, glucose tolerance and insulin sensitivity of *Nkcc1*^*β*^KO mice. **A, B**. Plasma insulin (A, pmol/L) and whole blood glucose (B, mg/dl) of 10w, 20w and 30w old *Nkcc1*^*βKO*^ and control (*Ins1*^*Cre*^) mice fed or fasted 16h. Results represent the mean ± SEM (*n* = 17–28, *p<0.05). **C, D**. Blood glucose excursions (mg/dl) during glucose tolerance tests (GTT, C) performed in 6h fasted *Nkcc1*^*βKO*^ and control (*Ins1*^*Cre*^) mice of the indicated ages (mean ± SEM, *n* = 9–14, *p<0.05) and the areas under the curve (D, mg/ml/min) of those responses. **E**. Blood glucose responses to exogenous insulin during insulin tolerance tests (ITT) performed in 6h fasted *Nkcc1*^*βKO*^ and control (*Ins1*^*Cre*^) mice at 10w, 20w and 30w of age. Each point represents the mean ± SEM (*n* = 9–16, *p<0.05).

Because 30w old *Nkcc1*^*βKO*^ mice developed NASH, we further tested glycemic control in these mice. Plasma insulin and blood glucose were determined in 10-30w old *Nkcc1*^*βKO*^ mice after their nocturnal feeding or after preventing it. Ten-week old *Nkcc1*^*βKO*^ showed minimal changes in fed or fasted plasma insulin and blood glucose levels relative to control mice (*Ins1*^*Cre*^, Fig 6A and 6B, *left panels*). However, fasting plasma insulin levels increased in 20w old *Nkcc1*^*βKO*^ mice and both, fed/fasted plasma insulin and blood glucose were significantly higher in *Nkcc1*^*βKO*^ mice at 30w of age (Fig 6A and 6B, *center* and *right panels*). Therefore, fasting hyperinsulinemia precedes the rise in blood glucose in *Nkcc1*^*βKO*^ mice whereas fed hyperinsulinemia and high blood glucose develop in older *Nkcc1*^*βKO*^ mice. Still, 30w old *Nkcc1*^*βKO*^ mice were not overtly hyperglycemic (*e*.*g*., >200 mg/dl) or hyperinsulinemic (*e*.*g*., >500 pmol/L) indicating that the secretory dysfunction/β-cell loss in islets lacking *Nkcc1* is insufficient to trigger T2D in chow-fed *Nkcc1*^*βKO*^ mice younger than ~35w. Instead, it results in age-dependent worsening of glycemic control. In support of that conclusion, 10w and 20w old *Nkcc1*^*βKO*^ mice were normo-tolerant to exogenous glucose (Fig 6C, *left and mid panel*), whereas 30w *Nkcc1*^*βKO*^ mice were not (Fig 6C, *right panel* and Fig 6D). In addition, 30w old *Nkcc1*^*βKO*^ mice developed resistance to insulin-induced hypoglycemia (Fig 6E, *right panel*). Therefore, the excess weight goes in hand with increased fasting plasma insulin but appears before overt glucose intolerance and insulin resistance in *Nkcc1*^*βKO*^ mice.

## Discussion

We present evidence indicating that *Nkcc1*^*βKO*^ mice develop a cluster of metabolic conditions compatible with the MetS. The metabolic features of *Nkcc1*^*βKO*^ mice are very similar to those found in the chow-fed *Fatzo/Pco* mouse model of MetS/NAFLD [67]. In fact, *Fatzo/Pco* mice became overweight/obese on a normal chow diet, independently of changes in energy intake [68] and had deficient insulin responses to oral glucose [67, 69]. Although the mechanisms responsible for the phenotypes of the *Fatzo/Pco* mouse model are complex and likely polygenic, the ones present in *Nkcc1*^*βKO*^ mice appear directly related to the functional loss of *Nkcc1* in β-cells. Indeed, *i*) *Cre*-mediated recombination of *"floxed"* alleles was only detected in β-cells of *Ins1*^*Cre*^:*Nkcc1*^*lox/lox*^:*Tomato* (Fig 1A–1H) and *Nkcc1*^*βKO*^ mice (Fig 1I–1L), respectively; *ii*) *Nkcc1*^*βKO*^ islets exhibited expected recombination events and *Nkcc1* transcript expression patterns (Fig 1M–1P); and *iii*) immunoreactive *Cre* was present only in β-cells of *Ins1*^*Cre*^ mice (S2I–S2P Fig). Importantly, the use of *Nkcc1* antibodies validated against knockout tissues (S1A–S1H Fig) showed the expected expression pattern of *Nkcc1* in tissues/cells of the normal *Nkcc1*^*lox/lox*^ mouse, including those in Sertoli and chromaffin cells (S2Q and S2R Fig) [70, 71], ductal/epithelial cells of the pancreas, intestine (S2S and S2T Fig) [72, 73] and brain in *Ins1*^*Cre*^:*Nkcc1*^*lox/lox*^:*Tomato* mouse model (S1E–S1I Fig) [74]. Along the same lines, *Nkcc1* protein expression was intact in the choroid plexus (S1I Fig), a relevant finding because low levels of *Ins1* gene activity were previously reported [75]. Moreover, since expression of *Cre* or "*floxed*" alleles did not alter *Nkcc1* tissue expression patterns (S1 and S2 Figs), together these results support the conclusion that *Nkcc1*^*βKO*^ mice have lost *Nkcc1* in β-cells, minimizing recent concerns related to the efficacy/efficiency of the *Ins1*^*Cre*^ line to eliminate target genes [76].

Consistent with the previous conclusion, the secretory function of 10w and 22w old *Nkcc1*^*βKO*^ islets is reduced by ~25% and ~50%, respectively (S2A and S3A Figs). Notably, disruption or chronic pharmacological inhibition of *Nkcc1* does not eliminate insulin responses to glucose [35, 44, 45, 62]. This is attributed to the fact that β-cells express a wide range of Cl^−^transporters and channels with potential overlapping and/or compensatory function, at least to some extent [39]. Nevertheless, our results suggest that β-cell *Nkcc2a* [45, 77] is minimally involved in the reduced secretory response of *Nkcc1*^*βKO*^ islets, because bumetanide did not reduce insulin secretion (S2A and S3A Figs). Along those lines, the participation of VRAC (*Lrrc8a-e*) [33, 34, 78] in the secretory phenotype of *Nkcc1*^*βKO*^ islets is expected to be limited because inhibition of *Nkcc1* impairs β-cell volume regulation and VRAC activation [33, 79]. Independent of the potential participation of the furosemide-sensitive *Kcc2* (*Slc12a5*) [80] or that of other Cl^−^transporters or channels in the secretory response of *Nkcc1*^*βKO*^ islets, our data suggest that loss of *Nkcc1* in β-cells results in a rather mild age-related secretory dysfunction. Further, the demonstration that 10w old *Nkcc1*^*βKO*^ islets were significantly smaller than control due to decreased β-cell number and volume (Fig 2B–2E) implies that the overall reduced *in vitro* secretory responses of these islets is also related, at least in part to their hypoplastic nature.

The mechanistic relationship between the loss of *Nkcc1* in β-cells and reduced β-cell number/volume/size is intriguing but not surprising. It has been demonstrated that *Nkcc1* participates in cell proliferation [81–87]. Actually, inhibition of *Nkcc1* reduced the proliferative capacity of excitable neuronal progenitor cells by dampening the electrical activity of ionotropic GABA receptors [88], which are Cl^−^channels, whereas their activation increased mouse and human β-cell mass [89, 90]. Our results demonstrating reduced number of β-cell clusters in the pancreas of 10-30w old *Nkcc1*^*βKO*^ mice (Fig 2F) support a role for *Nkcc1* as a potential regulator of progenitor cell proliferation, because these clusters are considered proto-islets [91]. In addition, our data demonstrating reduced cell volume in β-cells lacking *Nkcc1* (Fig 2C) are consistent with its role as a key regulator of mammalian cell volume [92] and, in particular, with biophysical [64, 79], pharmacological [40, 93, 94] and molecular [44] data directly implicating *Nkcc1* in the regulation of β-cell volume/size.

Therefore, the physiological metabolic consequences of losing *Nkcc1* in β-cells are potentially related to reduced β-cell mass/volume/size due to dysregulated [Cl^–^]_i_, altered Cl^−^channel-mediated electrical activity or a combination of both. In fact, inhibition of β-cell *Nkcc1* reduced glucose-induced β-cell electrical oscillations modulated by Cl^−^channels [29, 31] whereas isovolumetric circadian oscillations in [Cl^–^]_i_, determined by the activity of *Nkcc1*/*Kcc*, established the frequency of action potential firings in electrically excitable cells [95]. Regardless of the underlying mechanisms, the age-related metabolic consequences of altered pulsatile/circadian insulin release are multiple [16]. These include hepatic *Insr* down-regulation, reduced insulin signaling and development of insulin resistance [96], impaired glucose tolerance, BW gain, dyslipidemia, liver fat accumulation and increased risk of NAFLD/NASH [15, 97]. As we have shown, *Nkcc1*^*βKO*^ mice fed *ad libitum* a chow diet recapitulated most of the previous metabolic phenotypes in an age-dependent manner. At 10w of age, *Nkcc1*^*βKO*^ mice showed reduced hepatic *Insr* expression/signaling (Fig 3A and 3B), mild focal liver steatosis (Fig 5F) and reduced hepatic glycogen stores (S5G and S5H Fig) considered early metabolic manifestations of deficient insulin-mediated responses *in vivo* [98, 99]. Importantly, lean 10w old *Nkcc1*^*βKO*^ mice also showed increased plasma glycerol (Fig 5A), an early marker of lipolysis, altered triglyceride turnover [100, 101] and a predictor of glucose intolerance/T2D in humans [102]. Consistently, 30w old *Nkcc1*^*βKO*^ mice developed glucose intolerance (Fig 6C and 6D), systemic insulin resistance (Fig 6E) and had increased responses to alanine (Fig 3D), a substrate almost exclusively used by the liver for *de novo* gluconeogenesis [45]. Further, older *Nkcc1*^*βKO*^ mice had fasting/fed hyperinsulinemia (Fig 6A), hyperglycemia (Fig 6B) and developed overweight (Fig 4A–4C), severe dyslipidemia (Fig 5A and 5B) and NASH (Fig 5G and 5H and S5 Fig). Therefore, the age-dependent metabolic phenotype of *ad libitum* chow-fed *Nkcc1*^*βKO*^ mice resembles most of the natural history of metabolic syndrome. In a physiological setting, our data rises the possibility that β-cell *Nkcc1* may play a role in the natural decline of metabolic health associated with aging. In a clinical setting, our results may also provide a potential mechanism whereby chronic use of loop diuretics may worsen glucose homeostasis in patients with metabolic syndrome or susceptible to develop T2D.

In summary, our results demonstrate that the mild metabolic dysfunction of 10w old *Nkcc1*^*βKO*^ mice represents early phenotypic manifestations linked to a primary defect in β-cell function/proliferation/differentiation consequence of losing a diuretic-sensitive Cl^−^cotransporter. In addition, given that these phenotypes are not related to increased food intake, but precede the onset of overweight, it seems reasonable to conclude that the cascade of age-related metabolic manifestations observed in these mice develop in parallel with BW gain, likely increasing the risk of developing T2D later in life.

## Methods

### Animals and housing

The Animal Care and Use Committee of Wright State University approved all methods involving mice, which were carried out in accordance to relevant guidelines and regulations. Mice were congenic on the C57BL/6J genetic background and crossed for ~10 generations. Mice harboring *loxP* sites flanking exon 8–10 of the *Slc12a2* gene (*Nkcc1*^*lox/lox*^, provided by Dr. Christian A. Hübner, Jena University, Germany) were mated to *Ins1*^*Cre*^ mice [Jackson Labs stock 026801, B6(Cg)-*Ins1*^*tm1*.*1(cre)Thor*^/J] constitutively expressing *Cre* recombinase only in pancreatic β-cells [103] to generate *Ins1*^*Cre*^:*Nkcc1*^*lox/lox*^ mice (*Nkcc1*^*βKO*^). As control, we used the following homozygous mice: *Ins1*^*Cre*^, *Nkcc1*^*lox/lox*^, *Nkcc1*^*WT*^ (C57BL/6J) and the *tdTomato* reporter line [Jackson Labs stock 007909, B6.Cg-Gt(ROSA)26Sor^tm9(CAG-tdTomato)Hze^/J] to verify β-cell recombination of target alleles. In our hands, homozygous *Cre* expression in β-cells or the presence of *"floxed"* alleles in mice (*Ins1*^*Cre*^ and *Nkcc1*^*lox/lox*^) were not associated with changes in glucose homeostasis as determined by: basal/fed blood glucose, plasma insulin, and glucose and insulin tolerance, consistent with previous reports [103–105]. Mice had *ad libitum* access to water and a standard chow diet [Envigo, Teklad 22/5 Rodent Diet #8640 (3.0kCal/g, 54% carbohydrates, 29% proteins and 17% fats)], except when they were fasted. In that case, only water was provided. Housing conditions were set as 12:12h light (0630-1830h) and dark (1830-0630h) cycles with an ambient temperature of ~22ºC. Data presented here correspond to experiments performed using male mice from ~10 to ~35 weeks (w) of age housed in groups as recently described [106].

### Genotyping and RT-PCR

Mice were genotyped by using conventional PCR (Phire Tissue Direct PCR Master Mix, ThermoFisher Sci., #F170L) and genomic DNA from tail-clips or isolated islets to assess *Cre*-mediated recombination of *Nkcc1*^*lox*^ alleles [107]. Three sets of amplifying primers were used (5’-3’). Set 1: GCA ATT AAG TTT GGA GGT TCC TT (*Nkcc1*-w106/f220s) and TGG TGT GAA GGA ACA GTT GG (*Nkcc1*-w106/f220a). Set 2: GCA ATT AAG TTT GGA GGT TCC TT (*Nkcc1*-w206/f320s) and TGG TGT GAA GGA ACA GTT GG (*Nkcc1*-w206/f320a). Set 3: *Nkcc1*-w106/f220s, *Nkcc1*-w106/f220a and CCA ACA GTA TGC AGA CTC TC (*Nkcc1*-450r). Sets 1 and 2 amplify 106bp/206bp or 220bp/320bp from tail genomic DNA when mice are WT or carry *Nkcc1*^*lox*^ alleles, respectively. Set 3 was designed to co-detect recombined and *Nkcc1*^*lox*^ alleles as bands of 450bp and 206bp, respectively. Total RNA for RT-PCR experiments was obtained from freshly isolated mouse islets by using the RNeasy mini kit (Qiagen, Valencia, CA) reverse transcribed into cDNA (SuperScript II reverse transcriptase, ThermoFisher Sci., #18064022) and DNAse I-treated (New England Biolabs Inc., Ipswich, MA #M0303). Islet *Nkcc1* cDNAs were amplified by using the primer set 5’-ACA CCA CCA GCA GTA CTA CT-3’ (*Nkcc1*-400s) and 5’GGC CAT TGC TAT TAC GAC GA-3’ (*Nkcc1*-400) as previously done [80].

### Plasma biochemical studies, blood glucose and tolerance tests

Plasma was obtained after a 6h or 16h fasting period (0730-1330h or 1600-0800h, respectively) or at 0800h from mice fed *ad libitum*, by using heparinized glass capillaries (Scientific Glass, Rockwood, TN) and processed essentially as described [45]. Plasma triglycerides (TGs) and glycerol concentrations were determined by using commercially available kits (Cayman, Ann Harbor MI #10010303 and #10010755, respectively) and following the manufacturer’s instructions. Plasma insulin was quantified by using an ultrasensitive ELISA (10-1247-01; Mercodia, Winston-Salem, NC). Whole blood glucose was determined with a glucometer (FreeStyle-Lite, Abbott, IL). Glucose and insulin tolerance tests (GTTs and ITTs, respectively) consisted in measuring 6h fasted glucose and serially 15, 30, 60 and 120 minutes after intraperitoneal administration of 2.0g/kg D-glucose or 0.75U/kg of human recombinant insulin (HumulinR Eli Lilly, Indianapolis, IN). Alanine tolerance tests (ATTs) were performed in 16h fasted mice as described [45].

### Primary islets and insulin secretion

Mice were deeply and terminally anesthetized (Euthasol^®^, *ip* 150mg/kg) and pancreas tissues processed to isolate islets by using the collagenase method as previously described [45]. Islets were handpicked into individual wells of 12-well plates with mesh inserts [15 islet equivalents (iEq)/well] containing KRBH (in mM: 118.5 NaCl, 2.5 CaCl_2_, 1.2 KH_2_PO_4_, 4.7 KCl, 25 NaHCO_3_, 1.2 MgSO_4_, 10 HEPES and 0.1% BSA pH 7.4) plus 3.3mM glucose. The mesh inserts containing islets were transferred to new wells containing KRBH+3.3mM glucose and incubated at 37ºC (5% CO_2_) for 30 minutes, a step repeated once more. The islets were then transferred into their respective experimental wells containing KRBH+5.5mM or +12.5mM glucose plus vehicle (DMSO) or bumetanide (#B3023, Sigma Chem Co. Saint Louis, MO) for 1h at 37ºC (5% CO_2_). Islets were transferred into new wells containing KRBH+12.5mM glucose plus vehicle or drugs, incubated 1h at 37ºC (5% CO_2_) and transferred to new wells containing acidified ethanol. The KRBH from experimental wells was frozen at –20ºC for further analysis. Insulin content or secreted into the media was estimated using ELISA (10-1247-01, Mercodia, Salem, NC). Results are expressed as the ratio between secreted insulin and the sum of secreted and islet insulin content.

### Tissue processing and immunofluorescence microscopy

Mice were deeply anesthetized (Euthasol^®^, *ip* 150mg/kg), transcardially perfused with ice-cold PBS/heparin (0.1mM/1000U/ml, pH7.4) and then with ice-cold 4% paraformaldehyde (PFA) fixative to sacrifice them and collect tissues essentially as described [35]. Tissue embedding, sectioning and staining [(hematoxylin-eosin (H&E) and periodic acid-Schiff (PAS)] were done at AML Laboratories (Saint Augustine, FL). Additional tissue sections were processed for immunolabeling or H&E-staining (Sigma-Aldrich #HHS16 and 1% Phloxine B #19350 Certified Generon plus eosin Y #SE23-500D Fisher Chemical) and mounted (Permount SP15-100, Fisher Sci., Waltham MA) to capture digital images. To that end, we used a digital camera mounted on a Nikon Eclipse 600 microscope (Nikon Corp., Japan). Immunofluorescence microscopy experiments were performed as described [35] and the primary antibodies used were: *RFP* (rabbit, Rockland #35634), *Cre* (mouse, Millipore #MAB3120), *insulin* (guinea pig, Cell Marque #273A-15), *glucagon* (mouse, Abcam #K79bB10) and *somatostatin* (rat, Abcam #30788). We also used two KO-validated *Nkcc1* antibodies [rabbit, Abcam #59791 [108] and Aviva #OABB01332, S1 Fig]. Species-specific DyLight405/Cy3/AlexaFluor488-conjugated secondary antibodies were purchased from Jackson Immunoresearch Inc. (PA, USA). Note that DyLight405-conjugated secondary antibodies were used to visualize insulin-positive β-cells and that images were converted to gray-scale to increase contrast against Cy3/AlexaFluor488-conjugated antibody signals.

### Body composition and liver fat content analysis

Total body fat, lean mass and body water were determined in live mice by using the whole body quantitative magnetic resonance imaging (QMRI) analyzer EchoMRI-500^™^ system (EchoMRI LLC, Echo Medical Systems, Houston TX) as described [109]. Mice were then sacrificed by decapitation to determine hepatic fat content (w/w) by using the gravimetric method of Bligh and Dyer [110]. Briefly, liver samples were homogenized in chloroform:methanol:water (2:2:1.8) using a manual glass/glass homogenizer on ice. The homogenate was centrifuged at 625×g and the organic phase collected and washed once with double distilled water to help with phase separation. The chloroform phase containing extracted fat was vacuum-dried in a rotary evaporator (SC110A SpeedVac Plus) at high drying rate. The residue was then analytically weighed (Mettler Toledo, AE100).

### Tissue morphometry and histopathology analysis

Weighed pancreas tissues from mice were post-fixed, sectioned every 100μm and immunostained to assess endocrine cell areas (μm^2^), relative density (islet area/section area), endocrine cell number (counts/islet, only cells with a clear nucleus were counted), volume (pL, assuming spherical shape of cells) and mass [cell area per tissue section area × pancreas weight (g)]. We used NIH *Fiji* (*ImageJ* v2.3.0/1.53f, https://imagej.net/software/fiji/) [111] and digital images (1000dpi) taken at medium or high magnification (×400 to ×600, calibrated scale: 4.7–6.3 pixels/μm, respectively, corresponding to 0.045–0.024μm^2^/pixel^2^) using regular or oil immersion objectives (×60; Olympus Epi Fluorescence Spot Scope) attached to a color digital camera. Isolated, clusters of ≤5 β-cells or single β-cells within ductal epithelial cells were counted as identifiers of potential neogenic islets [91, 112]. Adipose tissue morphometry was performed by using the *Adiposoft* plug-in for *Fiji* (*imagej*.*net/plugins/adiposoft*) on H&E-stained retroperitoneal fat tissue sections at 100× and 200× magnification. Surface area data (pixels^2^) were manually transformed to μm^2^ after calibration against a 10μm ruler [1pixel^2^ = 0.754μm^2^ (100×) and 0.189μm^2^ (200×)]. The results were then confirmed by applying the automatic measuring function of the plug-in. Hepatic steatosis was blindly assessed by one of us (MDiF) and the preliminary diagnosis confirmed/extended by an experienced histopathologist (Dr. David Mirkin, Children’s Hospital, Dayton, OH). Images were subsequently analyzed to score steatosis severity by applying the criteria of Kleiner *et al*. [66]. These consist in the unweighted sum of three different scores: *i*) hepatocellular micro/macrovesicular steatosis (0, <5%; 1, >5–33%; 2, >33–66% and 3, >66% at 200×-400× magnification), *ii*) lobular inflammation as clusters of ≥5 inflammatory cells (0, no *foci*; 1, 1 *foci*/field; 2, 2 *foci*/field and 3, >2 *foci*/field at 100× magnification) and *iii*) cell ballooning (0, none; 1, few and 2, many cells with ballooning at 200×-400× magnification).

### Western blotting

Tissues were weighed and immediately submerged in liquid nitrogen or immediately processed to extract proteins. Briefly, liver tissues were minced and quickly homogenized at 4ºC in a glass/glass homogenizer (Wheaton 15ml) containing Radioimmunoprecipitation assay (RIPA) lysis buffer (Sigma, Saint Louis, MO, #R3792) supplemented with phenylmethylsulfonyl fluoride (PMSF) and a protease/phosphatase inhibitor cocktail (Thermo Sci., Waltham, MA, #78443) to a proportion of 3ml RIPA per gm of tissue. Tissue lysates were transferred onto a 15ml conical tube (Fisher Scientific, Corning #430790) and re-homogenized by passing the lysate ~20 times through 18-gauge needles attached to a 5ml plastic syringe followed by ~20 more strokes through 21-gauge needles. Protein concentration in tissue extracts was determined by using the Coomassie-Bradford protein assay kit (Thermo Sci., Waltham, MA, #23200) following the instructions of the manufacturer. Up to 50μg of total proteins boiled 5min in denaturing loading buffer (Novex #2107345) were resolved in duplicates by polyacrylamide gel electrophoresis (PAGE) by using Bolt^™^ 4–12%, Bis-Tris pre-casted gels (ThermoFisher Sci., #NW04120). Molecular weights were estimated by using pre-stained protein standards (SeeBlue Plus 2, ThermoFisher Sci., #LC5925). Gels were run at 130V for 35min in 2-(N-morpholino)-ethanesulfonic (MES) acid buffer (ThermoFisher Sci., #B000202), removed and soaked in 20% ethanol for 5 mins before transferring them onto pre-assembled transfer PVDF stacks (iBolt Transfer Stack, ThermoFisher Sci.). Proteins were electroblotted onto PDVF membranes by using a dry blotting system (Life Technologies, iBolt 2) and then incubated in blocking buffer (SuperBlock T20, ThermoFisher Sci. #37516) overnight at 4ºC. Membranes were washed three times for 10min in Tris-buffered saline (TBS) plus Tween 20 (TBST) and exposed to primary antibodies for 48h at 4ºC with gentle rocking. Membranes were then washed four times for 10min in TBST and exposed to relevant secondary antibodies for 1h at room temperature. After washing excess antibodies, antigen/antibody reactions were developed by chemiluminescence (Pierce West Pico Plus, ThermoFisher Sci., #34577). Images were taken using ChemiDoc Imaging System (Bio-Rad, Hercules, CA). Membranes were either stripped off the first antibody and reblotted, or new blots were produced when different antibodies were needed to detect proteins of similar molecular weight. The primary antibodies used were directed against: *insulin receptor β-subunit (Insr)*, the S/T protein kinase *Akt* and its active version p*Akt* phosphorylated in S^473^ (rabbit, Cell Signaling #3025, #9272 and #9271, respectively), the catalytic subunit of glucose-6-phosphatase *G6Pc* (rabbit, Abcam ab83690) and *β-actin* (mouse, Developmental Studies Hybridoma Bank #528068). Secondary HRP-conjugated antibodies used were: anti-rabbit IgG and anti-mouse IgM (Jackson Immunoresearch, PA, #711-035-152 and #315-035-049, respectively).

### Energy intake

Net 24h food intake was recorded in individually identifiable group-housed mice at 10w, 20w and 30w of age. Data was collected during 2 consecutive weeks after a week of acclimation in a metabolic cage equipped to record the feeding behavior of mice in real-time (Feed and Water intake activity monitor system HM-2, MBRose, Faaborg, Denmark). The overall settings, calibration and design of these experiments have been described in detail elsewhere [106]. The feeding microstructure/dynamics and ambulatory activity of *Nkcc1*^*βKO*^ and *Ins1*^*Cre*^ shall be reported in a forthcoming manuscript.

### Statistics

Results are represented as mean values ± SEM, with the number of individual points (*n*) indicated. Statistical significance for a *p* value <0.05 between groups was obtained by applying one-way or two-way analyses of variance (ANOVA), as appropriate, followed by the Tukey-Kramer *post-hoc* test. Statistical analyses were conducted by using GraphPad Prism v7 (GraphPad Software Inc., San Diego, CA, USA). Normal distribution and homogeneity of data variance were tested using Shapiro-Wilk and F-tests, respectively.

## Supporting information

S1 Fig Validation of *Nkcc1* antibodies and tissue expression pattern of the transporter. A-H. Representative pancreas sections from 4w old male mouse lacking *Nkcc1* in all tissues (*Nkcc1*^*KO*^, A-D) and 20w old C57BL/6J WT mouse (*Nkcc1*^*WT*^, E-H) co-immunolabeled against *Nkcc1* (A and E), insulin (*Ins*, C and G) and glucagon (*Gcg*, B and F) to demonstrate specificity of *Nkcc1* immunoreactivity in insulin-positive β-cells of normal *Nkcc1*^*WT*^ mice only. **I**. Representative sagittal brain section of a 15w old *Ins1*^*Cre*^;*Nkcc1*^*lox/lox*^;*Tomato* mouse immunolabeled against *Nkcc1* to demonstrate specific immunoreactivity in six regions of the brain: the granule cell layer (*gcl*), corpus callosum (*cc*), anterior commissure (*ac*), third lobule of cerebellar vermis (*cv3*) and in the choroid plexus epithelium of the 3^rd^ (*cp3*) and 4^th^ (*cp4*) ventricle. Bar represents 25μm.
(TIF)

S2 Fig Expression pattern of immunoreactive *Nkcc1* in the pancreas and tissues of normal *Ins1*^*Cre*^ and *Nkcc1*^*lox/lox*^ mice. Representative pancreas sections from a 15w old male mouse expressing both *Cre* alleles in β-cells (*Ins1*^*Cre*^, A-D and I, J) or homozygous for *Lox* alleles (*Nkcc1*^*lox/lox*^, E-H and M-P) co-immunolabeled against *Nkcc1* (A, E and Q-T), *Cre* (i and M), glucagon (*Gcg*, B, F, J and N) and insulin (*Ins*, C, G, K and O) to demonstrate conserved expression patterns of *Nkcc1* immunoreactivity in insulin-positive β-cells of the islets and in the indicated tissues. Bar represents 25μm. Representative sections of the indicated tissues (Q-T) dissected from 20-25w old *Nkcc1*^*βKO*^ mice immunolabeled against *Nkcc1* by using a KO-validated primary antibody (OABB01332). Nuclei were stained with 4′,6-diamidino-2-phenylindole (DAPI).
(TIF)

S3 Fig Secretory response of 10w old islets and α-cell morphometry analysis. **A**. Insulin secretory responses to low (5.5mM) and high (12.5mM) glucose of islets from 10w old *Nkcc1*^*βKO*^ and control mice (*Ins1*^*Cre*^) in the presence of vehicle (DMSO) or 10μM bumetanide (BTD), as indicated. Results are expressed as the mean ± SEM of insulin secreted relative to total islet insulin content (*n* = 3, *p<0.05). **B-F**. Shown are α-cell number (B, counts per islet), volume (C, pL), area (D, μm^2^; and E, % of pancreas section) and α-cell mass (F, g) corresponding to 10w and 30w old *Nkcc1*^*βKO*^ and control (*Nkcc1*^*lox/lox*^) mice. The results in B-D represent the mean ± SEM of data corresponding to >700 individual glucagon-stained islets found in 19–21 pancreas tissue sections obtained from male mice (*n* = 3) of the indicated genotypes. Each point in E, F represents mean values per tissue section. **G**. Shown is the mean islet α/β-cell ratio of *Nkcc1*^*βKO*^ and control mice at the indicated ages (*p<0.05). Results were obtained by dividing the data in B and that of Fig 2B.
(TIF)

S4 Fig Energy intake, body water content, white adipose tissue inflammation and ectopic fat accumulation. **A**. Normalized 24h food intake (kCal/gBW/day) of *Nkcc1*^*βKO*^ and control (*Ins1*^*Cre*^) mice recorded for 14 days. Data represents the mean ± SEM (*n* = 9–10, *p<0.05 *vs*. genotype, ^■^*p*<0.05 *vs*. age). **B, C**. Free water mass (B, g) representing bladder content and water in stomach/intestines of mice of the indicated genotypes/ages and total water mass (C, g): total water − free water / lean mass of mice (*n* = 9–10). **D, E** and **F, G**. Shown are H&E-stained retroperitoneal white adipose tissue (D, E) and pancreas (F, G) sections of 10w (D and F) and 30w old mice (E and G) of the indicated genotypes. The squares are shown at higher magnification in the images below. Bars indicate 50μm.
(TIF)

S5 Fig Histopathology confirmation of steatohepatitis in 30w old *Nkcc1*^*βKO*^ mice. **A, B**. Mild (A) and severe (B) inflammatory cell infiltration *foci* in relatively healthy liver tissue. **C, D**. Fat-degeneration of hepatocytes (arrowheads). **E, F**. Macrovesicular/balooning steatosis (arrowheads) consistent with a score of 3 in Kleiner’s scale. **G, H**. PAS-stained liver sections of control (*Nkcc1*^*lox/lox*^) and *Nkcc1*^*βKO*^ mice at 10w (G) and 30w of age (H) to demonstrate glycogen stores in hepatocytes. Scale bar represents 20μm.
(TIF)

S1 Raw images Original full-size PCR and RT-PCR gels. The red rectangles on top of gels A and B represent the cropped areas used to build Fig 1N and 1P (B), respectively, in the main text. **A**. Original gel of genomic PCR experiments using DNA (+DNA) or not (–DNA) as templates. Shown are amplified DNA fragments of expected sizes obtained by using the primer sets indicated in Fig 1M. Also shown are additional control reactions performed by using primers designed to amplify 123bp of genomic sequences corresponding to the *Slc12a5* gene. **B**. Original full-size RT-PCR gel showing bands of expected sizes corresponding to *Cre* (390bp) and *Nkcc1* transcripts (400bp) amplified from total RNA purified from *Nkcc1*^*βKO*^ (lanes *a* and *b*) or from *Nkcc1*^*lox/lox*^ islets (lane *c*). As negative control, water was used instead of total WT RNA (lane *d*).
(TIF)

S2 Raw images Original full-size Western blots. The red rectangles on top of each blot correspond to the cropped areas used to build Fig 3A and 3B in the main text.
(TIF)

S1 Datasets (XLSX)
