# Supplementary figures and images for: Loss of Slc12a2 specifically in pancreatic β-cells drives metabolic syndrome in mice
Source: PLoS One. 2022 Dec 29;17(12):e0279560. doi: 10.1371/journal.pone.0279560 (PMC9799326; doi:10.1371/journal.pone.0279560)

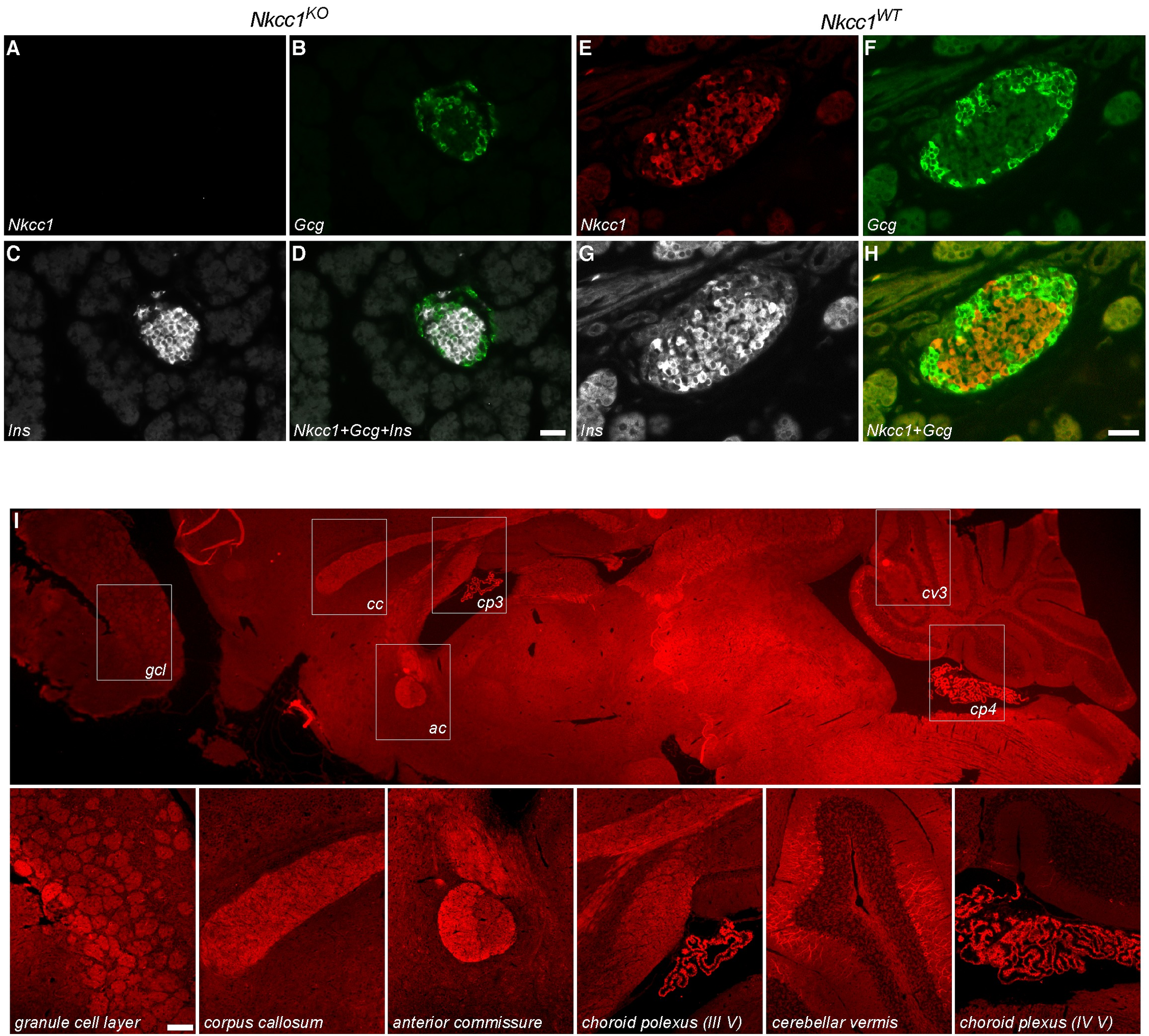

Supplement: S1 Fig — A-H. Representative pancreas sections from 4w old male mouse lacking Nkcc1 in all tissues (Nkcc1KO, A-D) and 20w old C57BL/6J WT mouse (Nkcc1WT, E-H) co-immunolabeled against Nkcc1 (A and E), insulin (Ins, C and G) and glucagon (Gcg, B and F) to demonstrate specificity of Nkcc1 immunoreactivity in insulin-positive β-cells of normal Nkcc1WT mice only. I. Representative sagittal brain section of a 15w old Ins1Cre;Nkcc1lox/lox;Tomato mouse immunolabeled against Nkcc1 to demonstrate specific immunoreactivity in six regions of the brain: the granule cell layer (gcl), corpus callosum (cc), anterior commissure (ac), third lobule of cerebellar vermis (cv3) and in the choroid plexus epithelium of the 3rd (cp3) and 4th (cp4) ventricle. Bar represents 25μm. (TIF) [file pone.0279560.s001.tif]

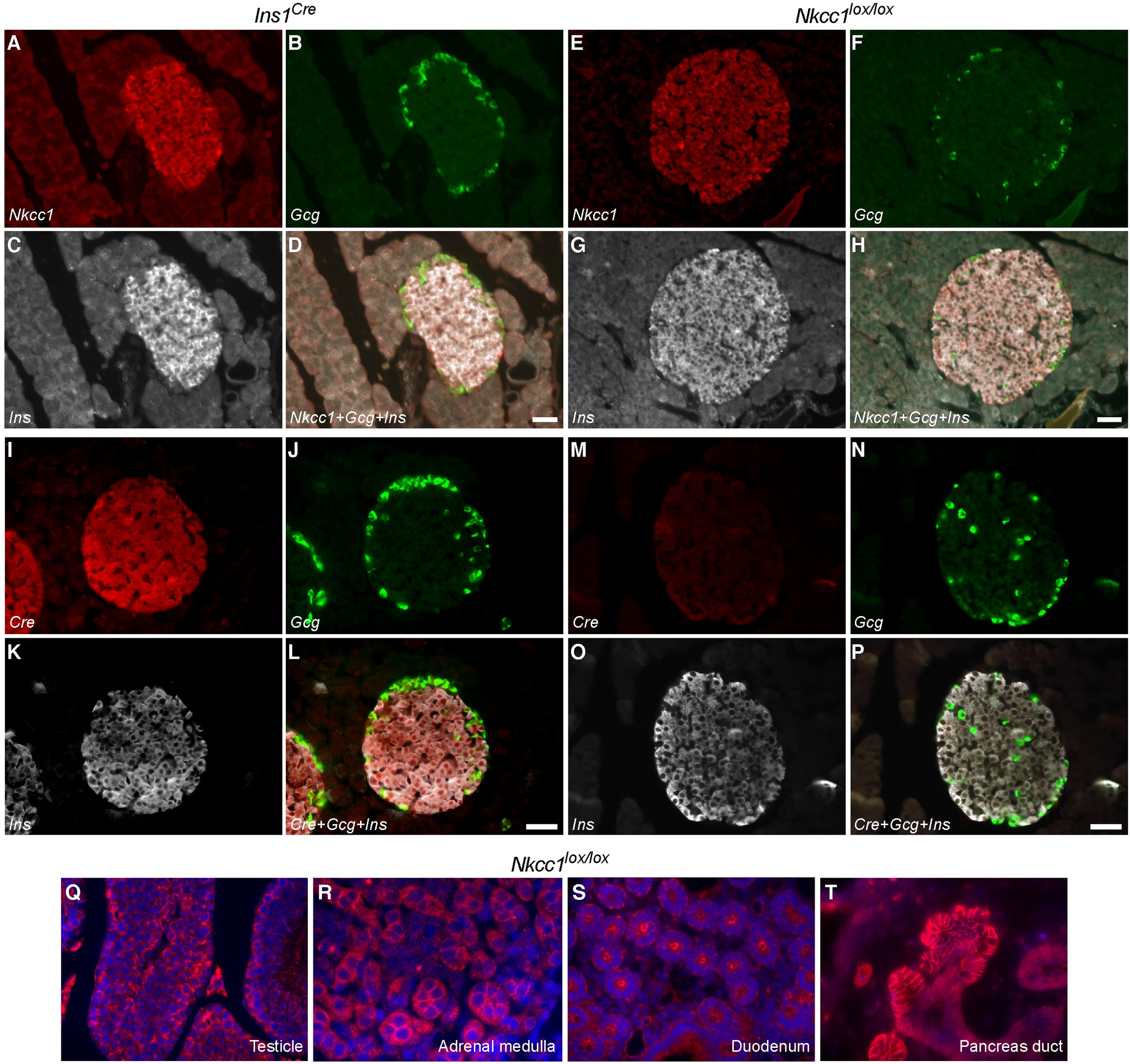

Supplement: S2 Fig — Representative pancreas sections from a 15w old male mouse expressing both Cre alleles in β-cells (Ins1Cre, A-D and I, J) or homozygous for Lox alleles (Nkcc1lox/lox, E-H and M-P) co-immunolabeled against Nkcc1 (A, E and Q-T), Cre (i and M), glucagon (Gcg, B, F, J and N) and insulin (Ins, C, G, K and O) to demonstrate conserved expression patterns of Nkcc1 immunoreactivity in insulin-positive β-cells of the islets and in the indicated tissues. Bar represents 25μm. Representative sections of the indicated tissues (Q-T) dissected from 20-25w old Nkcc1βKO mice immunolabeled against Nkcc1 by using a KO-validated primary antibody (OABB01332). Nuclei were stained with 4′,6-diamidino-2-phenylindole (DAPI). (TIF) [file pone.0279560.s002.tif]

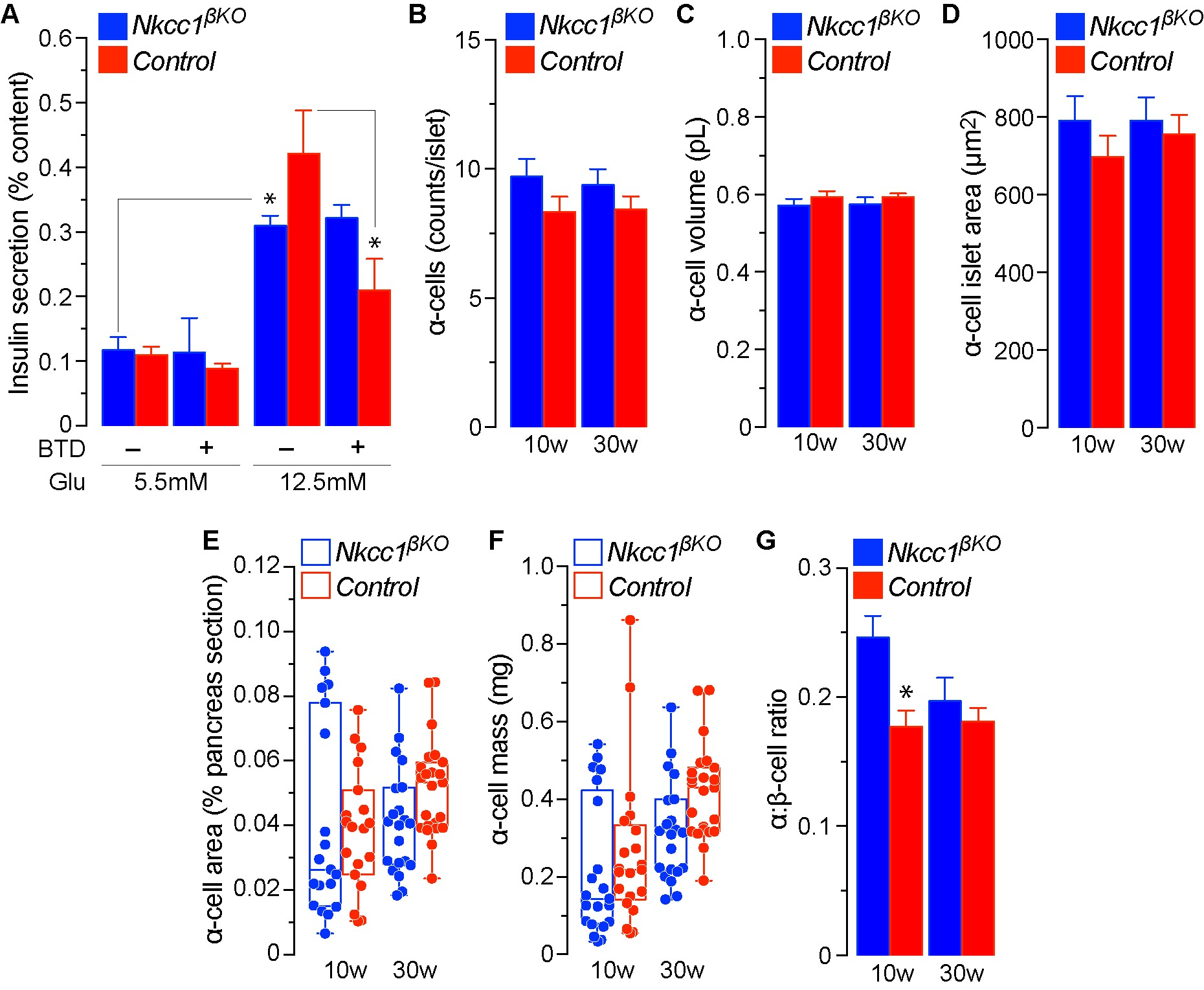

Supplement: S3 Fig — A. Insulin secretory responses to low (5.5mM) and high (12.5mM) glucose of islets from 10w old Nkcc1βKO and control mice (Ins1Cre) in the presence of vehicle (DMSO) or 10μM bumetanide (BTD), as indicated. Results are expressed as the mean ± SEM of insulin secreted relative to total islet insulin content (n = 3, *p<0.05). B-F. Shown are α-cell number (B, counts per islet), volume (C, pL), area (D, μm2; and E, % of pancreas section) and α-cell mass (F, g) corresponding to 10w and 30w old Nkcc1βKO and control (Nkcc1lox/lox) mice. The results in B-D represent the mean ± SEM of data corresponding to >700 individual glucagon-stained islets found in 19–21 pancreas tissue sections obtained from male mice (n = 3) of the indicated genotypes. Each point in E, F represents mean values per tissue section. G. Shown is the mean islet α/β-cell ratio of Nkcc1βKO and control mice at the indicated ages (*p<0.05). Results were obtained by dividing the data in B and that of Fig 2B. (TIF) [file pone.0279560.s003.tif]

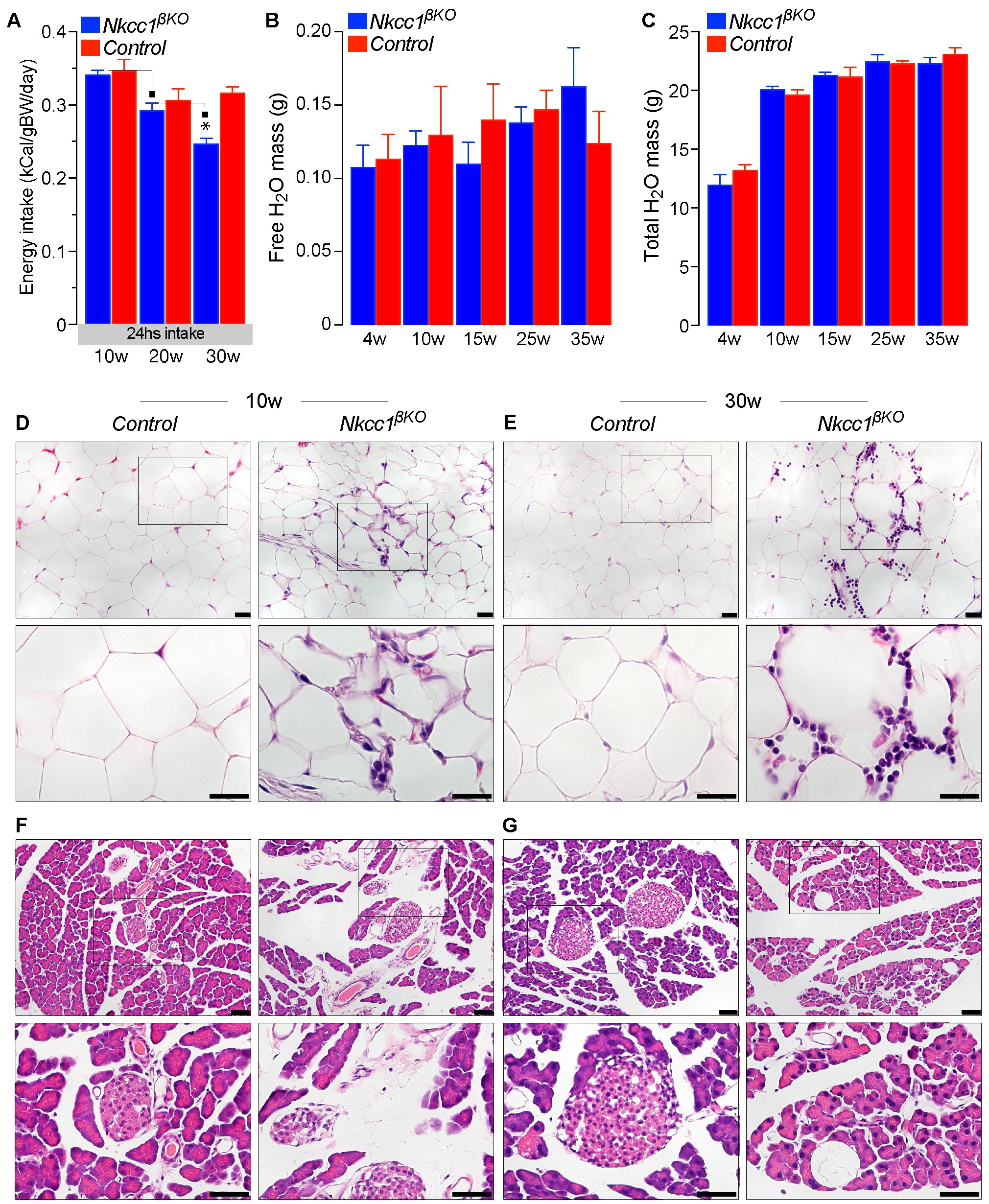

Supplement: S4 Fig — A. Normalized 24h food intake (kCal/gBW/day) of Nkcc1βKO and control (Ins1Cre) mice recorded for 14 days. Data represents the mean ± SEM (n = 9–10, *p<0.05 vs. genotype, ■p<0.05 vs. age). B, C. Free water mass (B, g) representing bladder content and water in stomach/intestines of mice of the indicated genotypes/ages and total water mass (C, g): total water − free water / lean mass of mice (n = 9–10). D, E and F, G. Shown are H&E-stained retroperitoneal white adipose tissue (D, E) and pancreas (F, G) sections of 10w (D and F) and 30w old mice (E and G) of the indicated genotypes. The squares are shown at higher magnification in the images below. Bars indicate 50μm. (TIF) [file pone.0279560.s004.tif]

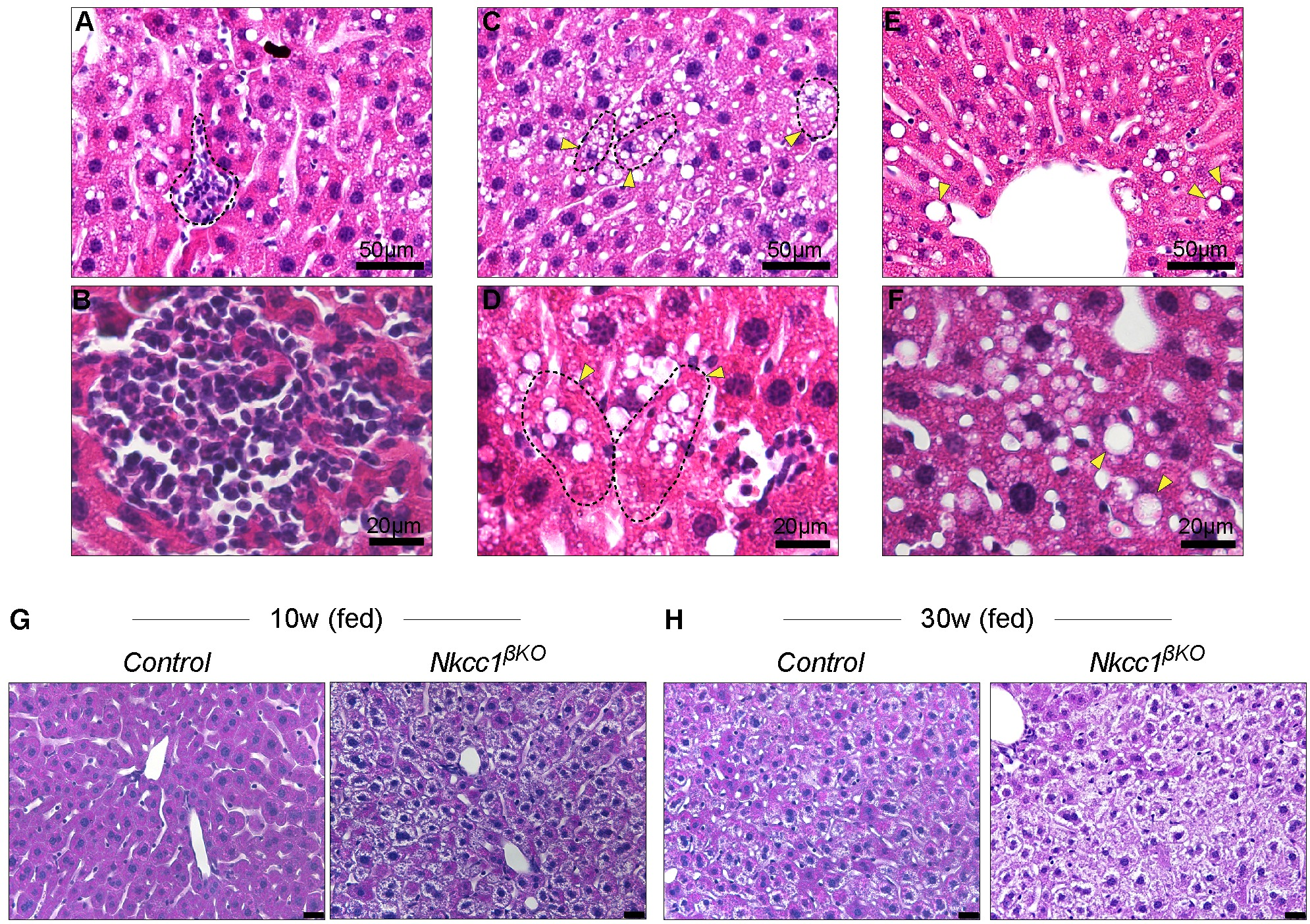

Supplement: S5 Fig — A, B. Mild (A) and severe (B) inflammatory cell infiltration foci in relatively healthy liver tissue. C, D. Fat-degeneration of hepatocytes (arrowheads). E, F. Macrovesicular/balooning steatosis (arrowheads) consistent with a score of 3 in Kleiner’s scale. G, H. PAS-stained liver sections of control (Nkcc1lox/lox) and Nkcc1βKO mice at 10w (G) and 30w of age (H) to demonstrate glycogen stores in hepatocytes. Scale bar represents 20μm. (TIF) [file pone.0279560.s005.tif]

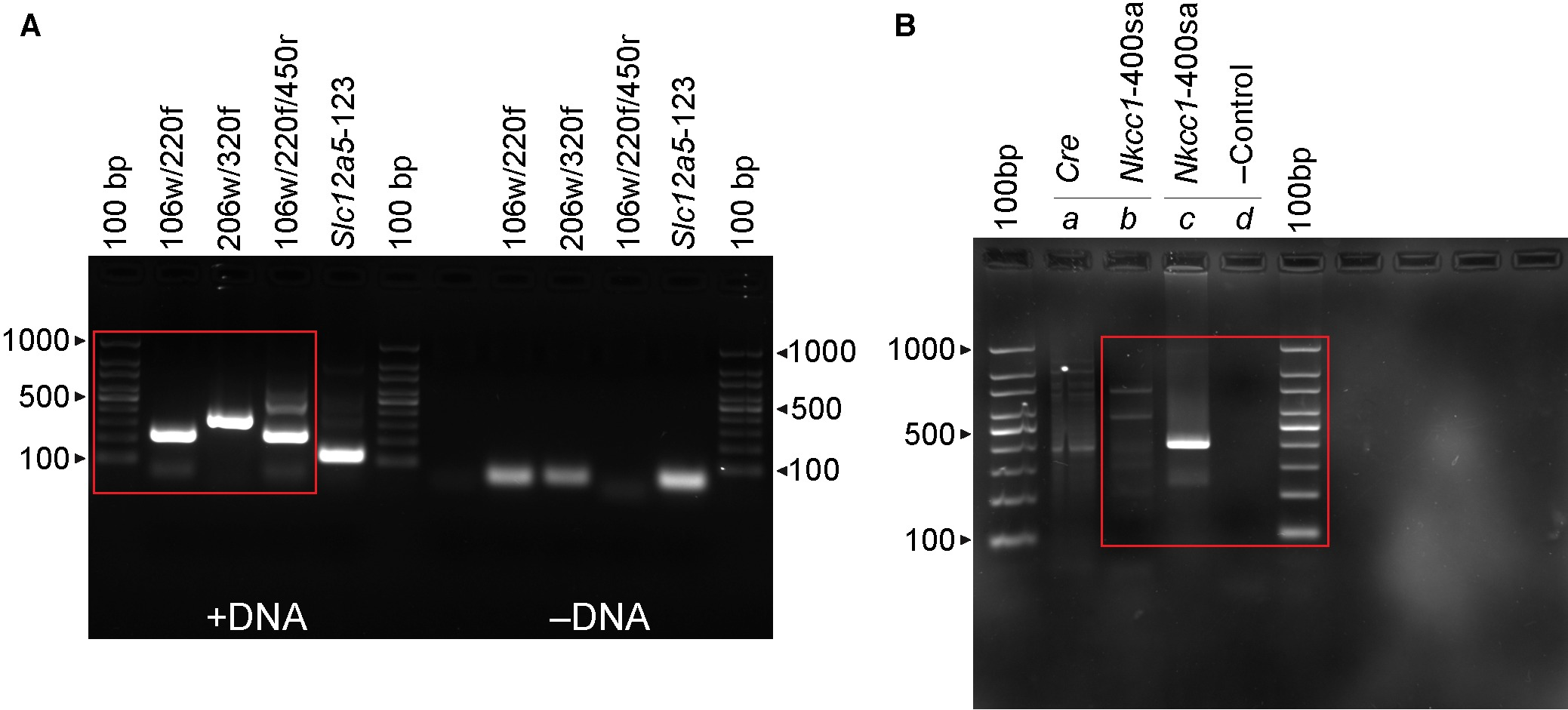

Supplement: S1 Raw images — The red rectangles on top of gels A and B represent the cropped areas used to build Fig 1N and 1P (B), respectively, in the main text. A. Original gel of genomic PCR experiments using DNA (+DNA) or not (–DNA) as templates. Shown are amplified DNA fragments of expected sizes obtained by using the primer sets indicated in Fig 1M. Also shown are additional control reactions performed by using primers designed to amplify 123bp of genomic sequences corresponding to the Slc12a5 gene. B. Original full-size RT-PCR gel showing bands of expected sizes corresponding to Cre (390bp) and Nkcc1 transcripts (400bp) amplified from total RNA purified from Nkcc1βKO (lanes a and b) or from Nkcc1lox/lox islets (lane c). As negative control, water was used instead of total WT RNA (lane d). (TIF) [file pone.0279560.s006.tif]

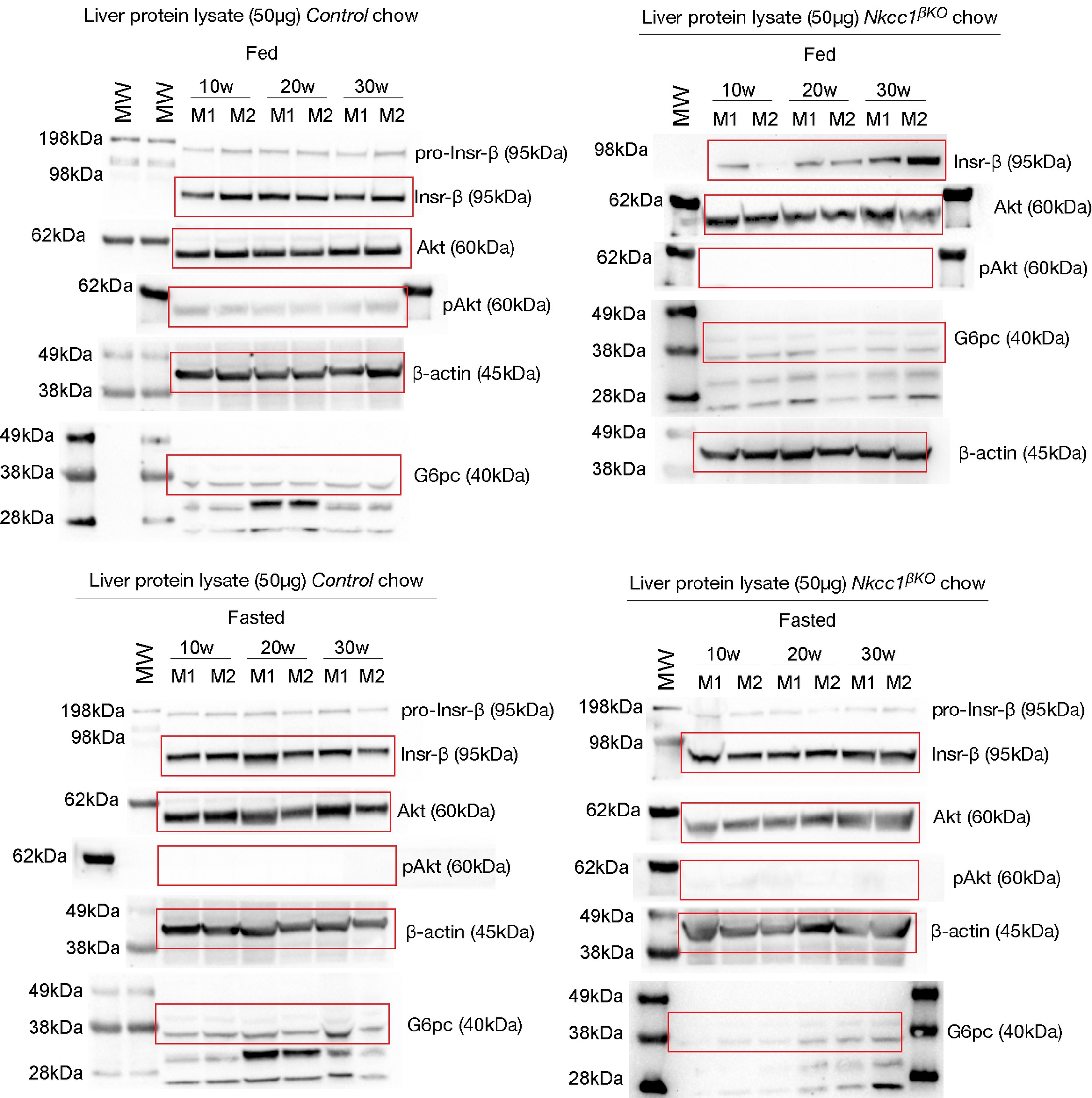

Supplement: S2 Raw images — The red rectangles on top of each blot correspond to the cropped areas used to build Fig 3A and 3B in the main text. (TIF) [file pone.0279560.s007.tif]
